# Lysosomal protease deficiency or substrate overload induces an oxidative-stress mediated STAT3-dependent pathway of lysosomal homeostasis

**DOI:** 10.1038/s41467-018-07741-6

**Published:** 2018-12-17

**Authors:** Jonathan Martínez-Fábregas, Alan Prescott, Sander van Kasteren, Deena Leslie Pedrioli, Irwin McLean, Anna Moles, Thomas Reinheckel, Valeria Poli, Colin Watts

**Affiliations:** 1 Division of Cell Signalling and Immunology, School of Life Sciences, University of Dundee, Dundee, DD1 5EH UK; 2 Division of Bio-Organic Chemistry, Leiden Institute of Chemistry, Einsteinweg 55, Leiden, 2333CC Netherlands; 3 Division of Molecular Medicine, School of Life Sciences, University of Dundee, Dundee, DD1 5EH UK; 4 Fibrosis Research Group, Institute of Cellular Medicine, Newcastle University, Newcastle upon Tyne, NE2 4HH UK; 5 Institute of Molecular Medicine and Cell Research, Medical Faculty, Albert-Ludwigs-University, Freiburg, D-79104 Germany; 6 Department of Genetics, Biology and Biochemistry, University of Turin, Via Nizza 52, 10126 Turin, Italy; 7 Present Address: Department of Molecular Mechanisms of Disease, University of Zurich, Winterthurestrasse190, 8057 Zurich Switzerland; 8 Institute of Biomedical Research of Barcelona, Spanish Research Council, Barcelona, 08036 Spain

## Abstract

Diverse cellular processes depend on the lysosomal protease system but how cells regulate lysosomal proteolytic capacity is only partly understood. We show here that cells can respond to protease/substrate imbalance in this compartment by de novo expression of multiple lysosomal hydrolases. This response, exemplified here either by loss of asparagine endopeptidase (AEP) or other lysosomal cysteine proteases, or by increased endocytic substrate load, is not dependent on the transcription factor EB (TFEB) but rather is triggered by STAT3 activation downstream of lysosomal oxidative stress. Similar lysosomal adaptations are seen in mice and cells expressing a constitutively active form of STAT3. Our results reveal how cells can increase lysosomal protease capacity under ‘fed’ rather than ‘starved’ conditions that activate the TFEB system. In addition, STAT3 activation due to lysosomal stress likely explains the hyperproliferative kidney disease and splenomegaly observed in AEP-deficient mice.

## Abstract (web summary)

How cells regulate their lysosomal proteolytic capacity is only partly understood. Here, the authors show that lysosomal protease deficiency or substrate overload induces lysosomal stress leading to activation of a STAT3-dependent, TFEB-independent pathway of lysosomal hydrolase expression.

## Introduction

Endosomes and lysosomes are now known to participate in multiple aspects of cell and tissue physiology besides their classical role in degradation of endocytosed substrates. They host key signalling systems such as the nucleic acid sensing Toll-like receptors (TLRs)^1,2^ and mTOR pathway^3,4^, and they generate immunological information through class II MHC-mediated antigen presentation^5,6^. Lysosomal proteases can drive a physiologically important caspase-independent cell death pathway^7,8^, and lysosome-like organelles allow cytotoxic leucocytes such as CD8 T cells, mast cells and eosinophils to execute their specific functions^9^. In addition, lysosomes are central to autophagy^10^. Most of these functions depend on protease activities found in the lysosomal lumen. Because these hydrolytic events are separated from the major cellular signalling pathways by the lysosomal membrane until recently it has not been obvious how a requirement for more or less hydrolytic capacity would be signalled to the cytosol and onwards to the transcriptional apparatus.

How lysosomal gene expression is controlled was advanced substantially by the identification of a signalling pathway that leads to the activation of transcription factor EB (TFEB)^11,12^. In response to cellular starvation, some types of lysosomal stress and some lysosomal storage diseases, TFEB translocated from the cytosol to the nucleus to drive the transcription of a variety of genes involved in lysosomal and autophagic function^3,13^. TFEB is negatively regulated by sequestration in the cytosol but in response to nutrient deprivation and reduced mTORC1 activity it becomes dephosphorylated, enters the nucleus and activates its target genes. In addition, a PKC-dependent but mTORC1-independent pathway for TFEB activation was recently described^14^. As important as this pathway is, there are reasons to suspect that additional regulatory mechanisms of lysosomal hydrolytic capacity may exist. For example, an increase in lysosomal protein substrate load and/or the accumulation of undegraded protein substrates is not expected to induce the TFEB pathway since this would be more consistent with a ‘fed’ rather than starved state. Nonetheless, increased hydrolytic capacity is likely needed to restore the status quo but how this would be achieved in the absence of mTOR/TFEB signalling is not clear.

Deletion of individual murine lysosomal proteases results in clear tissue-specific phenotypes illustrating that they have non-redundant functions^15–18^. Lysosomal proteolytic capacity relies mainly on three different enzyme families: the papain-like cysteine proteases (e.g. cathepsins B and L), the pepsin-related aspartyl proteases (cathepsins D and E) and a distinct cysteine protease known as asparaginyl endopeptidase (AEP) or legumain^19–21^. AEP shows high specificity for cleavage after asparagine^19,20^, suggesting it has specific processing functions. Consistent with this, AEP has been shown to make activating cleavages in TLR9 and TLR7 in dendritic cells^1,22^, and to participate in antigen processing^23^. AEP has also been recently linked to both exitoneurotoxicity and to neurofibrillary pathology through its site-specific cleavage of the DNAse inhibitor SET and tau, respectively^24,25^. Mice lacking AEP develop hyperproliferative kidney disease^26^ and several indications of hemophagocytic lymphohistiocytosis including hepatosplenomegaly^27^. How the absence of AEP triggers these hyperproliferative states is not known, but AEP is particularly abundant in the kidney proximal tubule^19,26^.

We demonstrate here a TFEB-independent, STAT3-dependent signalling pathway for lysosomal protease homoeostasis triggered by loss of lysosomal cysteine proteases or substrate overload. Chronic or acute AEP-deficiency promotes STAT3-dependent transcription of all three lysosomal protease families including AEP itself as well as other hydrolases. Lysosomal oxidative stress appears to be the primary driver of Jak2-STAT3 activation which is also observed in the AEP-deficient kidney proximal tubular system and which may explain the hyperproliferative disease and loss of kidney function in AEP-null mice.

## Results

### The lysosome detects loss of AEP activity and responds by increasing expression of multiple proteases

**Fig. 1:**
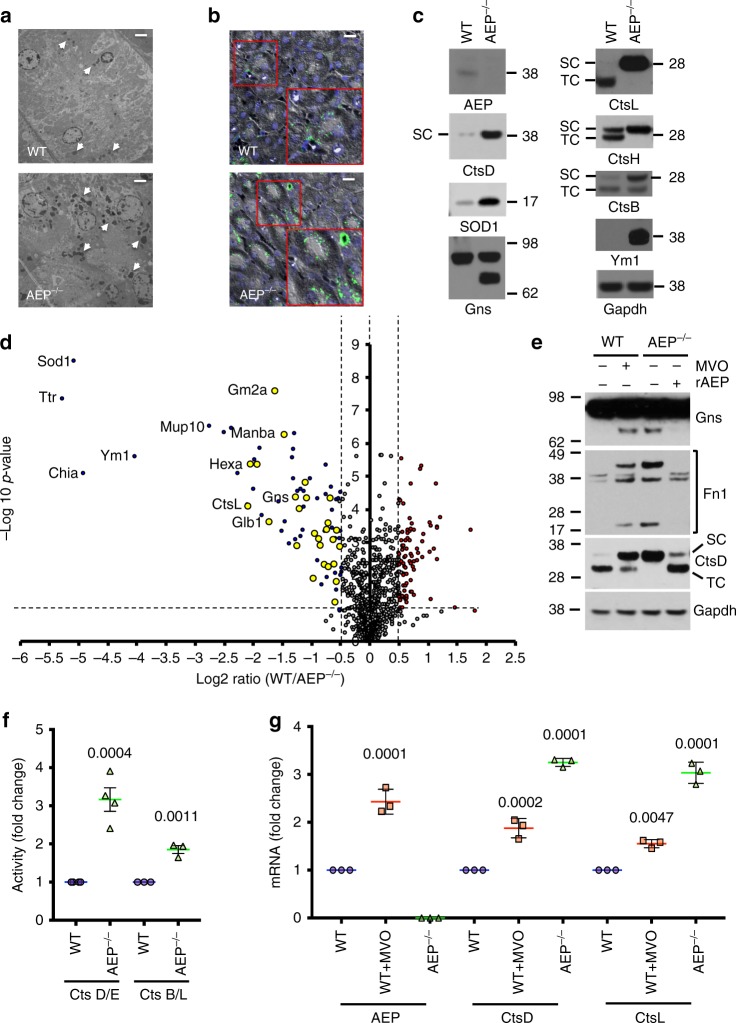
In vivo SILAC labelling reveals elevated lysosomal hydrolase expression induced by lack of AEP. **a** Transmission electron microscopy of WT (upper panel) and AEP^−/−^ (lower panel) kidney sections. Arrows indicate lysosomes. (Scale bar = 2 μm) **b** Immunofluorescence showing more prominent lysosomes (CtsD, green) in AEP^−/−^ kidney sections (lower panel) compared to WT kidney sections (upper panel). (Scale bar = 20 μm). **c** Western blot analysis confirming the accumulation in AEP^−/−^ kidneys of proteins identified by mass spectrometry. **d** Volcano plot showing proteins over-represented (blue dots, lysosomal proteins highlighted in yellow) or under-represented (red dots) in AEP^−/−^ lysosomal kidney fractions against the −log10 *p*-value for three independent experiments. Dotted line on *y* axis indicates *p* value < 0.05. **e** Acute AEP inhibition with MVO26630 recapitulates hydrolase induction in WT MEF and AEP reconstitution reverses it in AEP^−/−^ MEF. **f** Induction of CtsD/E and CtsB/L activities in AEP^−/−^ MEF compared to WT MEF. Data are the average ± SD of *n* = 4 biologically independent samples for CtsD/E or *n* = 3 biologically independent samples for CtsB/L. Statistical significance was calculated using a two-sided unpaired *t-*test. **g** Semi-quantitative RT-PCR analysis of mRNA levels for different cathepsins in WT MEF, WT MEF treated overnight with 50 μM MVO26630 and AEP^−/−^ MEF. Data are the average ± SD of *n* = 3 biologically independent samples. Statistical significance was calculated using a Dunnett’s multiple comparison test

Deletion of AEP in the murine kidney leads to proximal tubular cell (PTC) hyperplasia, interstitial fibrosis and eventually loss of kidney function^26^. At the morphological level lysosomes appeared more prominent in AEP-null kidney PTC and cathepsin D (CtsD) staining was more intense (Fig. 1a, b) indicating accumulation of undegraded material and/or increased expression of lysosomal proteins. Indeed, the levels of CtsL, which accumulates in its active single chain (SC) form when AEP is absent^26,28^, and other hydrolases such as the active single chain form of CtsD were higher in AEP-null kidney (Fig. 1c).

**Fig. 2:**
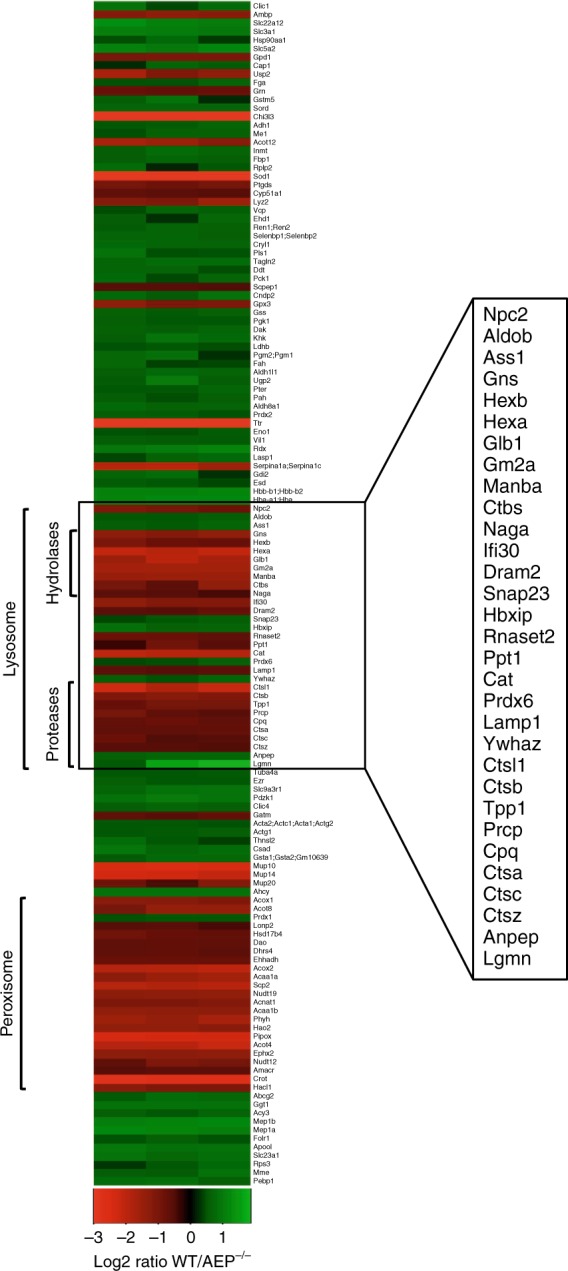
Lack of AEP induces an increase in the hydrolytic capacity of lysosomes in vivo. Heatmap built using heatmap.2 in the gplots package of R program showing proteins over-represented (red) or under-represented (green) in the AEP^−/−^ lysosomal kidney fractions compared to WT samples as identified by our SILAC approach, organised by cellular location and molecular function

We investigated this further using kidneys from mice fed a diet where ^12^C_6_-lysine is replaced by ^13^C_6_-lysine (‘SILAM mice’^29^) to reveal the proteome of age-matched wildtype (WT) and AEP-null kidney lysosomes (Supplementary Fig. 1a). In addition to proteins we previously detected by 2D-difference gel analysis^26^ including SOD1, transthyretin (Ttr) and Ym1 (Figs. 1c, d, 2 and Supplementary Data 1), mass spectrometric and bioinformatic analysis using the SILAM kidney as a reference sample revealed a striking increase in multiple proteins associated with lysosomes and peroxisomes in the AEP-null kidney (Figs. 1d, 2 and Supplementary Data 1) including cathepsins (Cts) A, B, C, L and Z and other hydrolases such as hexosaminidase A and hexosaminidase B, beta mannosidase (Manba) and ganglioside GM2 activator (Gm2a). We validated these changes by direct western blotting of WT and AEP-null kidney lysates (Fig. 1c). Although there was a modest increase in Lamp1 it appeared to be lysosomal contents rather than lysosomal membrane proteins that were increased. Although some differentially represented proteins may be undegraded AEP substrates, for example, fibronectin (Fn1)^30^, the general increase in hydrolase levels indicated an unexpectedly coordinated response to deletion of AEP.

This response was not specific to the kidney or to the in vivo state because many of the same proteins such as Gns and CtsD were elevated in SILAC-labelled AEP-null mouse embryonic fibroblasts (MEF; Fig. 1e and Supplementary Fig. 1b). Consistent with these changes, direct protease activity measurements in cell lysates (Fig. 1f) and in intact MEF (Supplementary Fig. 1c) confirmed enhanced CtsD/E and CtsB/L activity in AEP-null MEF. Moreover, the response could be induced acutely by treatment of WT MEF with the AEP inhibitor MVO26630, (hereafter MVO^31^) which led to increased Gns, CtsD and importantly AEP itself (Fig. 1e, Supplementary Fig. 2a, b and Supplementary Data 1). To assess reversibility of the phenotype, we reconstituted AEP-null MEF with pro-AEP which is taken up and converted to active AEP^32^. Restoration of AEP activity reversed the accumulations seen in AEP-null MEF (Fig. 1e). Increased protease levels was due to a transcriptional response because chemical AEP inhibition increased mRNA levels of AEP, CtsD and CtsL while genetic deletion of AEP induced even more substantial mRNA increases for CtsD and CtsL (Fig. 1g). Thus, loss of AEP activity promotes its own biosynthesis alongside several other lysosomal hydrolases.

### Increased lysosomal proteolytic capacity induced by suppression of AEP activity

**Fig. 3:**
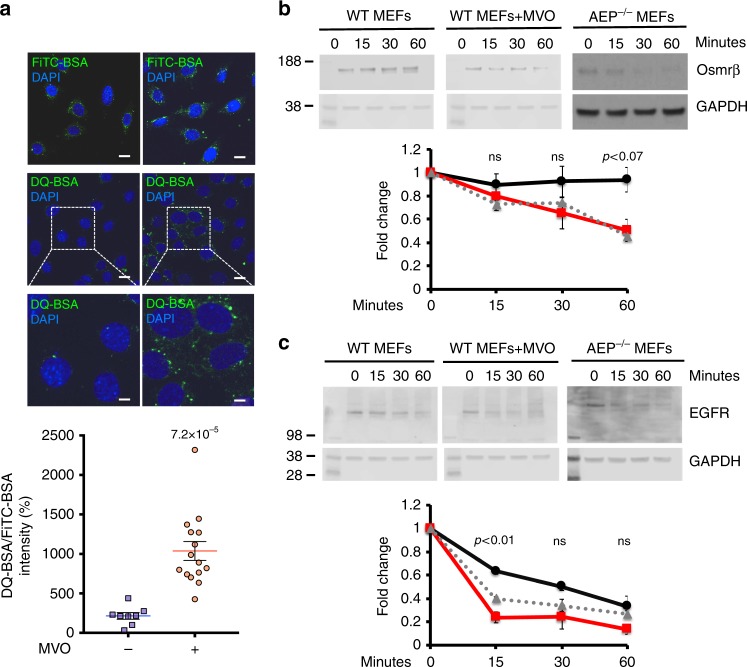
Increased lysosomal proteolytic capacity induced by suppression of AEP activity. **a** FiTC-BSA uptake and DQ-BSA degradation rates in WT 3T3s treated (red circles) or untreated (blue squares) with 50 μM MVO26630 for 16 h. Data are means ± SEM of *n* = 8 microscopic fields for untreated and *n* = 15 microscopic fields for MVO-treated. (Scale bar = 20 μm). **b** Time course of degradation of oncostatin M receptor beta (Osmrβ) in WT MEFs treated (dashed grey line) or untreated (black solid line) with 50 μM MVO26630 for 16 h and AEP^−/−^ MEFs (red solid line) following receptor downregulation by oncostatin M. Relative intensity of each Osmrβ band was calculated for three independent experiments. **c** Time course of degradation of epithelial growth factor receptor (EGFR) in WT MEFs treated (dashed grey line) or untreated (black solid line) with 50 μM MVO26630 for 16 h and AEP^−/−^ MEFs (red solid line) following receptor downregulation by epithelial growth factor. Relative intensity of each EGFR band was calculated for three independent experiments. Statistical significance was calculated using a two-sided unpaired *t-*test. ns = not significant

These results suggested an interesting and potentially important prediction: AEP ablation might actually lead to faster degradation of substrates traversing the endocytic pathway. We tested this for an exogenously supplied substrate and for two endogenous receptors. WT 3T3 fibroblasts, previously incubated with MVO to block AEP activity, were loaded with DQ-BSA, whose fluorescence is quenched prior to protease action. As shown in Fig. 3a, MVO-treated 3T3s showed stronger fluorescence compared with untreated 3T3s consistent with greater lysosomal protease capacity in the former. Moreover, ligand-induced downregulation of oncostatin M receptor beta (OSMRβ) and EGFR was faster in AEP-null and MVO-treated WT MEF compared with WT MEF (Fig. 3b, c). Thus, unexpectedly, genetic or chemical ablation of AEP elicits a response which, overall, leads to increased lysosomal proteolytic capacity.

### AEP suppression triggers a TFEB-independent increase in lysosomal hydrolase expression

**Fig. 4:**
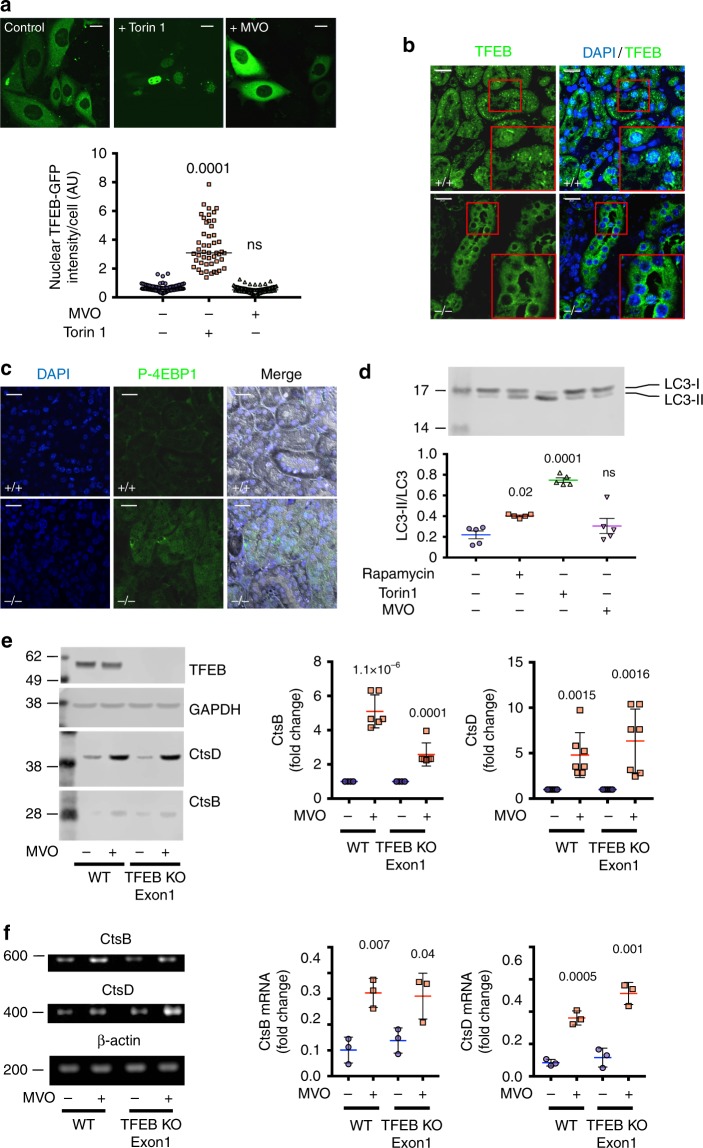
Increased lysosomal hydrolytic capacity induced by chronic or acute AEP-deficiency does not depend on TFEB activation. **a** Cellular localisation of TFEB-GFP in HKC-8 cells treated or untreated with 50 μM MVO26630 or 1 μM Torin1 overnight. (Scale bar = 20 μm). Data are the average ± SD of *n* = 93 cells for control sample, *n* = 48 for Torin1-treated sample and *n* = 92 for MVO-treated sample. Statistical significance was calculated using a Dunnett’s multiple comparison test. **b** Localisation of TFEB by IF in WT and AEP^−/−^ kidney sections. (Scale bar = 20 μm). **c** Levels of P-4EBP1 by IF in WT and AEP^−/−^ kidney sections. (Scale bar = 20 μm). **d** Immunoblotting and quantitation of LC3II/total LC3 ratio in lysates from WT MEFs treated with Rapamycin (1 μM) or Torin1 (1 μM) for 4 h or with MVO26630 (50 μM) for 16 h. Data are the average ± SD of *n* = 5 biologically independent samples. Statistical significance was calculated using a Dunnett’s multiple comparison test. **e** Levels of expression of CtsB and CtsD in HKC-8 WT and TFEB knockout Exon 1 treated or untreated with 50 μM MVO26630 for 16 h. Data are the average ± SD of *n* = 6 biologically independent samples for CtsB and *n* = 7 biologically independent samples for CtsD. Statistical significance was calculated using a two-sided unpaired *t-*test. **f** mRNA expression levels for CtsB and CtsD in HKC-8 WT and TFEB knockout Exon 1 treated or untreated with 50 μM MVO26630 for 16 h. Data are the average ± SD of *n* = 3 biologically independent samples. Full data and gel calibration markers can be seen in Supplementary Fig. 3c. Statistical significance was calculated using a two-sided unpaired *t-*test

We performed various experiments to test whether the upregulation of lysosomal hydrolases following AEP inhibition was due to a TFEB response^11,13^. First, we generated cells expressing TFEB-GFP and challenged them either with MVO or with the mTORC1 inhibitor Torin1. For these and some subsequent experiments, we utilised the human kidney PTC line, HKC-8^33^. As previously reported^34^, TFEB-GFP showed a striking redistribution from cytosol to nucleus upon mTORC1 inhibition (Fig. 4a). In contrast, TFEB remained cytosolic in MVO-treated MEF (Fig. 4a) and in AEP-null kidney PTC (Fig. 4b). In fact, in the latter case TFEB seemed to be excluded from the nucleus to a greater extent compared to WT PTC. This suggested elevated mTORC1 activity and indeed phospho-4EBP1 levels were higher in AEP-null kidney (Fig. 4c). Reduced mTORC1 activity leads to TFEB activation and downstream induction of autophagy genes^13^. To test further for a TFEB response when AEP was ablated, we examined the autophagy marker LC3 (microtubule-associated protein 1 light chain 3) in MVO, Torin1 or rapamycin-treated MEF. As expected, inhibition of mTORC1 induced a conversion of LC3-I to the phosphatidyl ethanolamine-conjugated form LC3II consistent with increased autophagic flux. In contrast, LC3 status, and therefore autophagy, was unchanged upon AEP inhibition (Fig. 4d). To investigate this apparent TFEB-independent response further, we used CRISPR-Cas9 technology to eliminate TFEB expression in HKC-8 cells (Supplementary Fig. 3a). As expected, upon starvation TFEB-null cells no longer induced an increase in cathepsin expression (Supplementary Fig. 3b). In contrast, when the same TFEB-null cells were treated with MVO, there was still a robust increase in expression of CtsD and CtsB at both protein (Fig. 4e) and mRNA (Fig. 4f and Supplementary Fig. 3c) levels. Thus increased lysosomal hydrolytic capacity did not in this instance involve a TFEB response.

### Activation of STAT3 in AEP-deficient cells

**Fig. 5:**
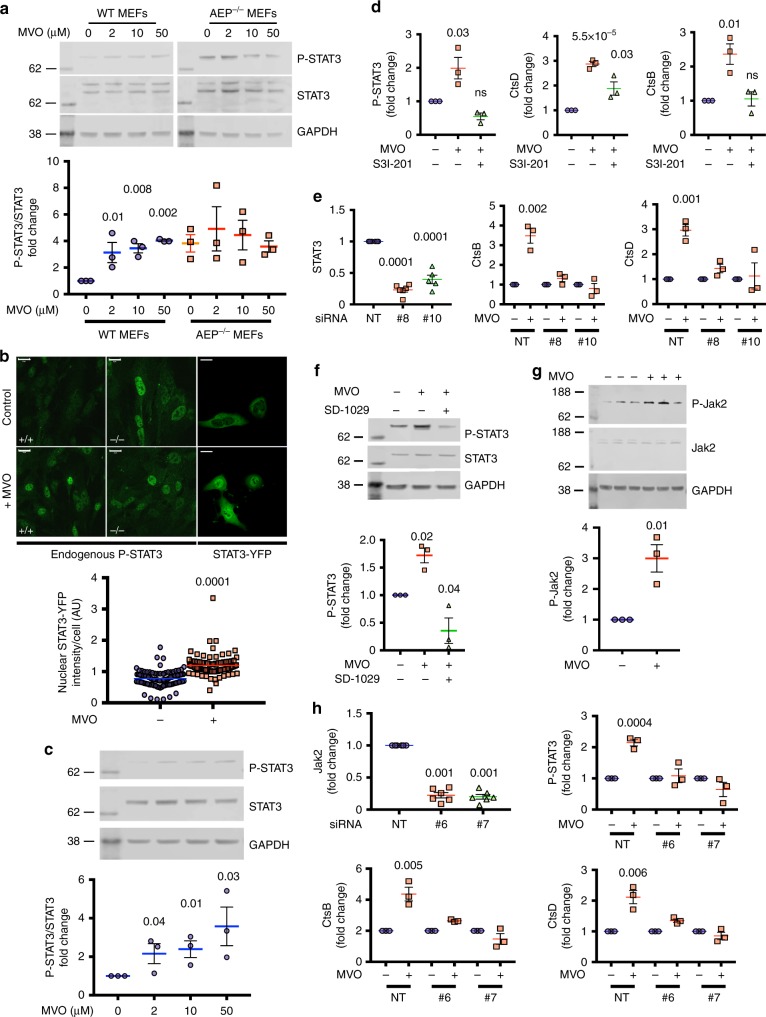
Increased lysosomal hydrolytic capacity induced by chronic or acute AEP-deficiency is controlled by the Jak2-STAT3 signalling pathway. **a** STAT3 activation in WT but not AEP^−/−^ MEFs treated for 16 h with increasing concentrations of MVO26630. Data are the average ± SD of *n* = 3 biologically independent samples. Statistical significance was calculated using a Dunnett’s multiple comparison test. **b** P-STAT3 localisation in WT and AEP^−/−^ MEFs treated or not with 50 μM MVO26630 for 16 h (endogenous P-STAT3) and HKC-8 cells transfected with STAT3-YFP and treated or not with 50 μM MVO26630 for 16 h. Data are the average ± SEM of *n* = 96 cells for control samples and *n* = 86 for MVO-treated samples. Statistical significance was calculated using a two-sided unpaired *t-*test. (Scale bar = 20μm). **c** STAT3 activation in HKC-8 cells treated with increasing levels of MVO26630 overnight. Data are the average ± SD of *n* = 3 biologically independent samples. Statistical significance was calculated using a two-sided unpaired *t-*test. **d** CtsD, CtsB and P-STAT3 levels in WT MEFs compared to WT MEFs treated overnight with 50 μM MVO26630 with or without 100 μM of the STAT3 inhibitor S3I-201. Data are the average ± SD of *n* = 3 biologically independent samples. Statistical significance was calculated using a two-sided unpaired *t-*test. **e** Levels of STAT3, CtsB and CtsD in WT HKC-8 cells transfected with a non-targeting siRNA (NT) or two different STAT3-targeting siRNAs (#8 and #10) and treated or not with 50 μM MVO26630 for 16 h. Data are the average ± SD of *n* ≥ 3. Statistical significance was calculated using a two-sided unpaired *t-*test. **f** P-STAT3 western blot in WT HKC-8 treated with 50 μM MVO26630 in the presence or absence of the Jak2 inhibitor SD-1029 (10 μM). Data are the average ± SD of *n* = 3 biologically independent samples. Statistical significance was calculated using a Dunnett’s multiple comparison test. **g** Levels of P-Jak2 in WT HKC-8 cells treated or untreated with 50 μM MVO26630 for 16 h. Data are the average ± SD of *n* = 3 biologically independent samples. Statistical significance was calculated using a two-sided unpaired *t-*test. **h** Levels of Jak2, P-STAT3, CtsB and CtsD in WT HKC-8 cells transfected with a non-targeting siRNA (NT) or two different Jak2-targeting siRNAs (#6 and #7) and treated or not with 50 μM MVO26630 for 16 h. Data are the average ± SD of *n* ≥ 3 biologically independent samples. Statistical significance was calculated using a two-sided unpaired *t-*test

Further analysis of cytosolic fractions from SILAC-labelled AEP-null MEF revealed a consistent increase both in the level and activation state of the transcription factor STAT3 (Fig. 5a, Supplementary Fig. 4a and Supplementary Data 1). Moreover, inhibition of AEP in WT MEF with MVO or with two other mechanistically distinct AEP inhibitors^35^ induced a dose-dependent increase in activated STAT3 (phospho-Tyr705-STAT3, hereafter P-STAT3) without affecting the levels of total STAT3 (Fig. 5a and Supplementary Fig. 4b). Importantly, P-STAT3 levels were already elevated in AEP-null MEF and were not further increased by MVO demonstrating that STAT3 was activated specifically by suppression of AEP activity (Fig. 5a). Phosphorylation of STAT3 on Tyr705 results in dimerisation and translocation into the nucleus^36^. P-STAT3 was found in the nuclei of AEP-null MEF and MVO-treated WT MEF but not untreated MEF (Fig. 5b). Similarly, in HKC-8 expressing STAT3-YFP, suppression of AEP activity clearly triggered nuclear translocation of STAT3 (Fig. 5b). MVO-treatment of HKC-8 cells also induced a dose-dependent increase in P-STAT3 (Fig. 5c).

### STAT3 mediates the lysosomal response to loss of AEP activity

The notion that STAT3 rather than TFEB might control lysosomal hydrolase expression under some conditions seemed plausible since a STAT3-dependent increase in the expression of several cathepsins during the involution of the mammalian mammary gland was recently shown^7,8^. We took multiple approaches to establish a link between STAT3 activation and lysosomal hydrolase expression in our system. Small molecule targeting of STAT3 with S3I-201^37^ not only blocked STAT3 phosphorylation but almost completely reversed the MVO-induced increase in CtsD and CtsB levels in MEF (Fig. 5d and Supplementary Fig. 5a). To confirm these results, we generated STAT3-depleted HKC-8 cells by siRNA knockdown (Fig. 5e and Supplementary Fig. 5b). Knockdown of STAT3 by two different siRNAs suppressed the MVO-induced increase in CtsB and CtsD protein levels, a result not seen with a non-targeting siRNA control (Fig. 5e and Supplementary Fig. 5b). Further, we also generated a STAT3 mutant (fused to GFP to distinguish it from endogenous STAT3) designed to resist siRNA knockdown (mSTAT3-GFP). MVO-induced induction of CtsD was rescued in cells expressing mSTAT3-GFP, which in contrast to endogenous STAT3, was largely resistant to siRNA knockdown (Supplementary Fig. 5c) demonstrating unequivocally a requirement for STAT3. Consistent with canonical activation of STAT3, inhibition of Jak2 with the inhibitor SD-1029 suppressed MVO-induced STAT3 phosphorylation (Fig. 5f and Supplementary Fig. 5d). Moreover, MVO treatement of HKC-8 cells activated Jak2, as indicated by enhanced phosphorylation on Tyr1007/1008 (Fig. 5g). To confirm the involvement of Jak2, we generated Jak2-depleted cells. As shown in Fig. 5h and Supplementary Fig. 5e, two different siRNAs directed to Jak2, but not a non-targeting siRNA, suppressed MVO-induced STAT3 phosphorylation as well as increased levels of CtsB and CtsD protein (Fig. 5h and Supplementary Fig. 5e). Thus, ablation of AEP activates the canonical Jak2-STAT3 pathway and leads to upregulation of other lysosomal hydrolases.

### Direct promotion of lysosomal hydrolase expression by STAT3

**Fig. 6:**
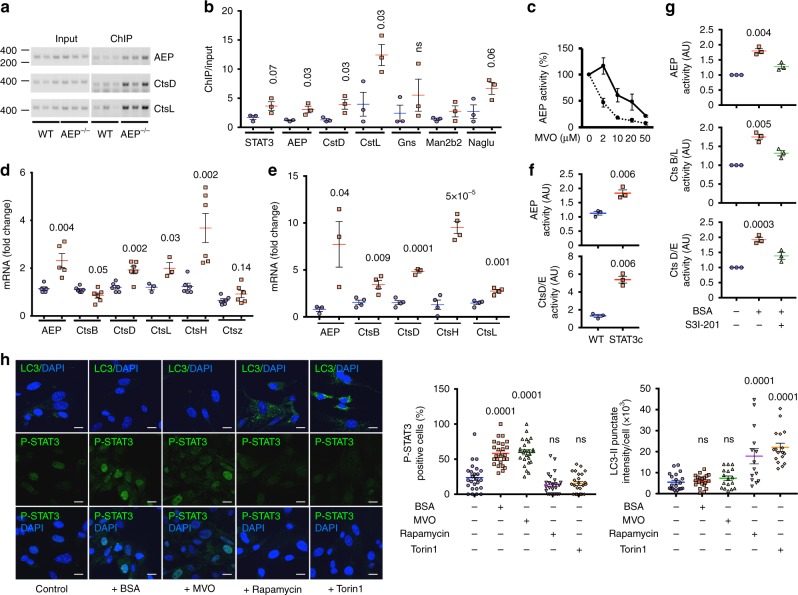
Direct STAT3 involvement in regulation of lysosomal hydrolase genes under conditions of protease activity/lysosomal substrate imbalance. **a** Agarose gel analysis of representative ChIP-PCR products for AEP, CtsD and CtsL promoters in WT and AEP-deficient MEFs. Full data and gel calibration markers can be seen in Supplementary Fig. 6. **b** Quantitative PCR analysis of DNA from STAT3 ChIP of WT (blue circles) and AEP-deficient (red squares) MEFs expressed as fold enrichment. Data are the average ± SD of *n* = 3 biologically independent samples. Statistical significance was calculated using a two-sided unpaired *t-*test. **c** AEP activity in WT MEF treated overnight with different concentrations of MVO26630 in the presence (dashed line) or absence (solid line) of 100 μM S3I-201. Data are the average ± SD of three independent experiments. **d** Quantitative PCR analysis of the mRNA levels for different lysosomal proteases in WT (blue circles) and STAT3c 3T3 (red squares) cells. Data are the average ± SD of *n* = 6 biologically independent samples for all samples, except for AEP where *n* = 5 and CtsL where *n* = 3. Statistical significance was calculated using a two-sided unpaired *t-*test. **e** Quantitative PCR analysis of the mRNA levels for different lysosomal proteases in WT MEFs treated (red squares) or not (blue circles) with 30 mg/ml BSA overnight. Data are the average ± SD of *n* = 4 biologically independent samples for all samples, except for AEP and CtsD where *n* = 3. Statistical significance was calculated using a two-sided unpaired *t-*test. **f** AEP and CtsD/E activities measured in WT and STAT3c 3T3 cells. Data are the average ± SD of *n* = 3 biologically independent samples. Statistical significance was calculated using a two-sided unpaired *t*-test. **g** Activities of AEP, CtsB/L and CtsD/E in WT MEF treated or not with 30 mg/ml BSA overnight in the presence or absence of 100 μM S3I-201. Data are the average ± SD of *n* = 3 biologically independent samples. **h** Quantitation of P-STAT3 and LC3II levels in WT MEFs treated with Rapamycin (1 μM) or Torin1 (1 μM) for 4 h or with MVO26630 (50 μM) or BSA (30 mg/ml) for 16 h. Data are the average ± SD of *n* = 25 microscopic fields for P-STAT3 and *n* = 14 for LC3II. Statistical significance was calculated using a Dunnett’s multiple comparison test. ns = not significant. (Scale bar = 20μm)

Overall, 67% of proteins increased 1.5-fold or more in AEP-null MEFs featured a canonical STAT3 site in their promoters while almost 90% contained either canonical or non-canonical STAT3 sites as defined in a recent study^38^. Although this was consistent with STAT3 recruitment to lysosomal hydrolase promoters it was important to test this directly in AEP^−/−^ versus WT cells. We immunoprecipitated STAT3-associated chromatin fragments (ChIP) from WT and AEP^−/−^ MEF and compared the signal obtained by PCR from input versus precipitated DNA for key upregulated hydrolase genes. We observed enhanced STAT3 recruitment to the promoters of CtsD, CtsL, the still intact promoter region of the AEP gene and other hydrolase genes in AEP^−/−^ compared with WT cells (Fig. 6a, b and Supplementary Fig. 6a). STAT3 recruitment to the AEP promoter made sense of the initially puzzling observation that low levels of MVO increased, rather than decreased AEP activity in cells. Blockade of STAT3 with S3I-201 prevented this increase and increased MVO potency at all concentrations (Fig. 6c). Taken together, these results demonstrate direct involvement of STAT3 in regulation of lysosomal hydrolase expression when a key lysosomal hydrolase activity is missing. Importantly, elevated activity of AEP and CtsD was also observed in 3T3 fibroblasts (Fig. 6f) expressing a constitutively active form of STAT3, STAT3c, which dimerises in the absence of tyrosine phosphorylation^39,40^, an increase which was again due to increased protease mRNA levels in these cells (Fig. 6d and Supplementary Fig. 6b).

### Protein overload induces STAT3-dependent protease expression

If the response triggered by AEP ablation is due to accumulation of undegraded substrates, we reasoned that a similar response should be induced by substrate overload of the endocytic pathway. To test this WT MEFs were incubated for 16 h with 30 mg/ml BSA and the status of STAT3 as well as protease mRNA levels and activities assessed. Protein overload led to a two to sevenfold increase in the mRNA levels of several lysosomal proteases including AEP, CtsB, CtsL, CtsD and CtsH (Fig. 6e and Supplementary Fig. 7) consistent with an increase in protease activity levels (Fig. 6g). This increase was blunted by STAT3 inhibition with S3I-201 (Fig. 6g). Importantly, increased protein load also led to a striking increase in nuclear P-STAT3 similar to that seen upon MVO-treatment (Fig. 6h) but without nuclear translocation of TFEB (Supplementary Fig. 8a). In contrast, mTORC1 inhibition with either rapamycin or Torin1 had no effect on P-STAT3 distribution but instead triggered focalised LC3 accumulation on autophagosomes, a response that was not observed either with MVO-treatment or protein overload (Fig. 6h). This important result demonstrates that STAT3-driven expression of lysosomal hydrolases is not a limited response to the perturbations caused by loss of AEP activity but one induced by increased load on the lysosomal system and therefore likely to be of widespread physiological significance. Moreover, the protease/substrate imbalance conditions that activate STAT3 do not signal through the mTORC1/TFEB pathway and conversely, suppression of mTORC1 activity which activates TFEB does not activate STAT3 (Fig. 6h).

### Lysosomal stress triggers STAT3-dependent de novo protease expression

**Fig. 7:**
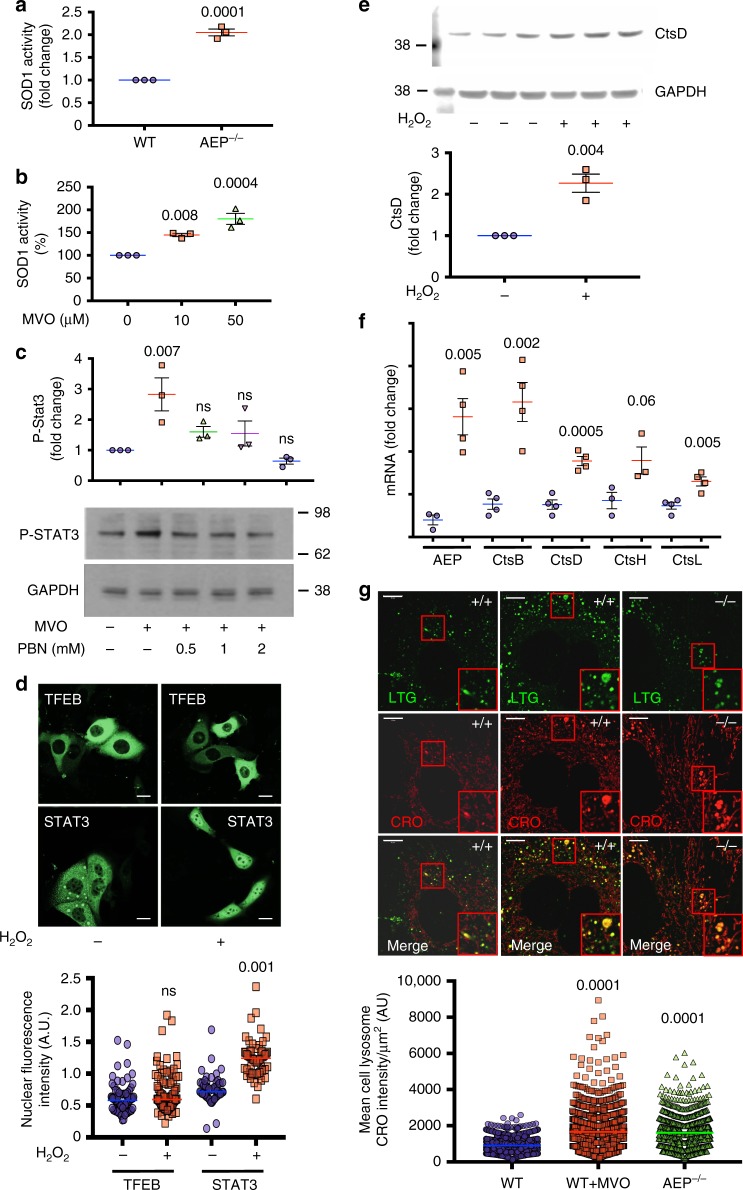
Reactive oxygen species: At the nexus between lysosomal hydrolytic capacity and STAT3 phosphorylation. **a** Superoxide dismutase 1 (SOD1) activity in WT and AEP^−/−^ MEFs. Data are the average ± SD of *n* = 3 biologically independent samples. Statistical significance was calculated using a two-sided unpaired *t-*test. **b** SOD1 activity in WT MEFs treated for 16 h with increasing concentrations of MVO26630. Data are the average ± SD of *n* = 3 biologically independent samples. Statistical significance was calculated using a Dunnett’s multiple comparison test. **c** MVO26630-induced (6 h, 50 μM) STAT3 activation in the presence of increasing concentrations of alpha Phenyl-*N*-*Tert* Butyl Nitrone (PBN). Data are the average ± SD of *n* = 3 biologically independent samples. Statistical significance was calculated using a Dunnett’s multiple comparison test. **d** Cellular distribution of TFEB-GFP (upper panels) and STAT3-YFP (lower panels) in HKC-8 WT treated or untreated with 0.2 mM H_2_O_2_ for 16 h. Quantitative data show the nuclear fluorescence intensity per cell (*n* = 79 for TFEB control, *n* = 99 for TFEB + H_2_O_2_, *n* = 59 for STAT3 control and *n* = 58 for STAT3 + H_2_O_2_). Statistical significance was calculated using a two-sided unpaired *t-*test. (Scale bar = 20μm). **e** Levels of CtsD in HKC-8 WT cells treated with 0.2 mM H_2_O_2_ for 16 h. Data are the average ± SD of *n* = 3 biologically independent samples. Statistical significance was calculated using a two-sided unpaired *t*-test. **f** Quantitative PCR analysis of the mRNA levels encoding several different lysosomal proteases in WT MEFs treated or not with 0.2 mM H_2_O_2_ overnight. Data are the average ± SD of *n* = 4 biologically independent samples for all samples, except for CtsH where *n* = 3. Statistical significance was calculated using a two-sided unpaired *t-*test. **g** Live cell imaging of lysosomal production of reactive oxygen species using CellROX Orange (CRO) in AEP^−/−^ or WT MEFs treated or not with 50 μM MVO26630 for 16 h. LysoTracker Green DND26 (LTG) was used as a lysosomal marker. Data represent the mean CRO intensity per lysosome normalised to lysosomal size in µm^2^ (*n* = 1254 lysosomes for WT MEFs sample, *n* = 1413 lysosomes for WT MEFs + MVO sample and *n* = 1204 for AEP MEFs). (Scale bar = 10μm). Statistical significance was calculated using a Dunnett’s multiple comparison test

STAT3 can be activated by a variety of exogenous and endogenous stimuli including oxidative stress^41,42^. We postulated that oxidative stress following acute AEP inhibition might be a driver of STAT3 activation since oxidative stress is a common feature of lysosomal storage diseases^43^. Consistent with this scenario, SOD1 levels were elevated in both AEP-null kidney^26^ and MEF (Fig. 1c, d and Fig. 7a), and SOD1 activity was also boosted following acute AEP inhibition with MVO (Fig. 7b). We strengthened the link between AEP-deficiency, oxidative stress and STAT3 activation in three ways. First, the ‘spin trap’ antioxidant phenyl-α-*tert* butyl nitrone (PBN) completely blocked the activation of STAT3 (Fig. 7c). Second, direct exposure to H_2_O_2_ induced the nuclear translocation of STAT3-YFP but not TFEB-GFP in HKC-8 cells (Fig. 7d) and moreover, increased the level of CtsD (Fig. 7e). Indeed, in H_2_O_2_ treated MEF, increased mRNA levels for multiple lysosomal proteases were observed (Fig. 7f and Supplementary Fig. 7). Third, CellROX Orange, a reporter dye that fluoresces upon oxidation showed a twofold increase in lysosomal labelling in AEP-null MEF and in WT MEF treated with MVO compared to untreated WT MEFs (Fig. 7g), thus identifying lysosomes as sites of ROS generation. Importantly, protein overload of lysosomes also induced oxidative stress signals in lysosomes (Supplementary Fig. 8b), underlining that STAT3 activation by lysosomal stress is a general response aimed at recovering lysosomal homoeostasis induced by lysosomal protease/substrate imbalance.

### AEP ablation is linked to STAT3 activation in murine kidney PTC in vivo and in human PTC

**Fig. 8:**
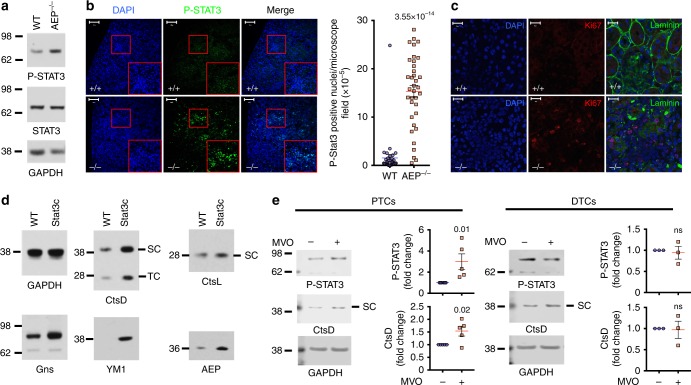
Activation of STAT3 in AEP^−/−^ kidney and enhanced hydrolase expression in STAT3c kidneys. **a** STAT3 and P-STAT3 levels in WT and AEP^−/−^ kidneys. **b** P-STAT3 detected by IF in WT and AEP^−/−^ kidney sections. Right panel shows quantitation of the number of P-STAT3 positive nuclei per area (*n* = 35 microscopic fields). (Scale bar = 100μm). Statistical significance was calculated using a two-sided unpaired *t-*test. **c** Ki67 and laminin detected by IF in WT and AEP^−/−^ kidney sections. (Scale bar = 20μm). **d** Similar proteins accumulate in STAT3c and AEP^−/−^ kidney. **e** Levels of P-STAT3 and CtsD in proximal (PTC) and distal (DTC) tubular cells treated or not with 50 μM MVO26630 overnight. Data are the average ± SD of *n* = 5 biologically independent samples for PTCs and *n* = 3 for DTCs. Statistical significance was calculated using a two-sided unpaired *t-*test

To test the physiological relevance of the STAT3-driven response to lysosomal stress, we re-examined kidneys from AEP-null mice and mice expressing constitutively activated STAT3c. Indeed, levels of P-STAT3 were higher in AEP-null kidney compared with WT although levels of STAT3 protein appeared similar (Fig. 8a). Importantly, labelling for P-STAT3 was particularly prominent in the PTC, which exhibits the highest levels of AEP expression^26^ (Fig. 8b). This finding most likely explains the hyperproliferative PTC phenotype observed in AEP-null kidney^26^ and confirmed here using anti-Ki67 staining (Fig. 8c). Once again, increased expression of lysosomal hydrolases could be uncoupled from AEP status since kidneys from STAT3c mice^44^ also showed striking increases in many of the proteins that were induced by AEP-deficiency or protein overload including CtsD and CtsL, Ym1, Gns and AEP (Fig. 8d). To test whether the response was conserved in primary human kidney cells, we expanded PTC as well as distal tubular cells (DTC) from biopsied human kidneys. MVO-treatment induced STAT3 activation and increased expression of CtsD in primary PTC but not in DTC (Fig. 8e).

### STAT3 regulation of lysosomal capacity in cathepsin depleted cells and tissues

**Fig. 9:**
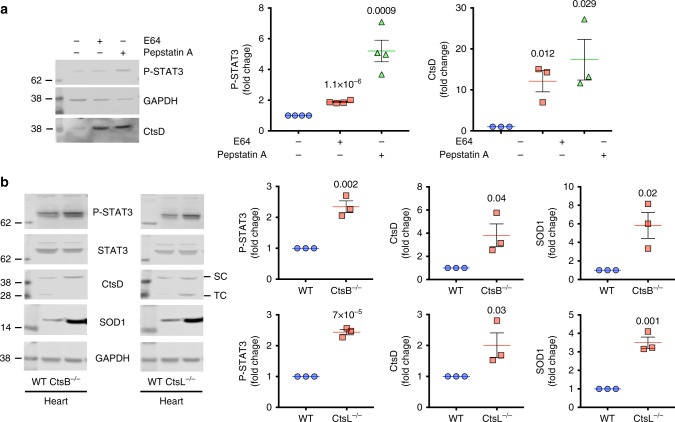
Chronic or acute inhibition of cysteine or aspartyl cathepsins lead to a similar response. **a** Levels of P-STAT3 and CtsD in WT MEFs treated or not with 10 μM E64 or 5 μM Pepstatin A for 72 h. Data are the average ± SD of *n* = 4 biologically independent samples for P-STAT3 and *n* = 3 biologically independent samples for CtsD. Statistical significance was calculated using a two-sided unpaired *t-*test. **b** Levels of P-STAT3, STAT3, CtsD and SOD1 in CtsB^−/−^ heart or CtsL^−/−^ heart compared to WT heart. Data are the average ± SD of *n* = 3 biologically independent samples. Statistical significance was calculated using a two-sided unpaired *t-*test

Finally, we addressed two further important questions: does ablation of lysosomal proteases besides AEP induce STAT3-driven hydrolase expression and do tissues other than the kidney show evidence of this response? First, we looked for an acute in vitro response akin to that induced by MVO by treating WT MEF with the cysteine cathepsin inhibitor E64 or the aspartyl cathepsin inhibitor pepstatin. WT MEF treatment with pepstatin increased P-STAT3 levels and boosted CtsD expression substantially as did E64 albeit to a lesser extent (Fig. 9a). Next, we examined tissues from mice genetically ablated for CtsL or B which are both E64 targets. We examined heart since previous studies had demonstrated a cardiomyopathic phenotype in ageing CtsL-null mice and enlarged lysosomes in the myocardium^45^. In CtsL-null heart (Fig. 9b), P-STAT3 and CtsD levels were increased by at least a factor of 2 (Fig. 9b). Similar increases were observed in CtsB-null heart tissue although no overt heart phenotype has been described in these mice. Moreover, as in cells and tissues lacking AEP, SOD1 expression was also increased in Cts-null tissues providing evidence that oxidative stress was a factor once again (Fig. 9b). Thus, ablation of diverse lysosomal protease activities can induce a STAT3-regulated response whose purpose is presumably to restore homoeostasis.

## Discussion

How cells tune the performance of their lysosomes to meet the demand placed on them has only recently been investigated. A particular challenge is to understand how the environment in the lysosomal lumen is sensed and communicated to cytosolic signalling pathways and the transcriptional machinery. Our results show that when the capacity of the lysosome to ‘keep up’ with substrate load is compromised, either because of loss of a key hydrolytic activity or because of increased endocytic load, oxidative stress leads to activation of STAT3 and the de novo transcription of a variety of lysosomal hydrolases. Although the endpoint is similar, this pathway differs fundamentally from the recently elucidated mTORC1-TFEB pathway for lysosomal biogenesis^46^.

A mechanism for enhancing lysosomal capacity independent of the mTORC1-TFEB pathway makes sense from several standpoints. In particular, the cellular response to nutrient poor conditions that promote autophagy would be expected to be different to the response to conditions which can be characterised as ‘nutrient rich’ where exogenous proteins are abundant. Recent studies have shown that cells can use bulk endocytic uptake pathways such as macropinocytosis to capture proteins and degrade them to meet nutritional demands and to drive cell proliferation^47,48^. Under these conditions, enhanced generation of amino acids in the lysosomal lumen would be expected to maintain or even enhance mTORC1 activity precluding TFEB activation. Indeed, most likely as a result of enhanced lysosomal protein breakdown and amino acid availability, we observed tight retention of TFEB on lysosomes under conditions that induced STAT3 activation.

Although ‘nutrient poor’ and ‘protein nutrient rich’ conditions both benefit from enhanced lysosomal capacity, other aspects of cellular physiology in these states are quite different. For example, a number of TFEB target genes are involved in autophagy^13^. Induction of autophagy alongside increased lysosomal capacity makes sense under nutrient poor conditions but probably not under conditions of increased substrate load since this would likely overload and stress the lysosomal system still further. In fact, faced with lysosomal overload of undegraded substrates, it would make sense to temporarily inhibit constitutive autophagy which is ongoing in most cells^49^. Consistent with this, there is extensive evidence that autophagy is indeed inhibited by STAT3, for example through an inhibitory interaction between protein kinase R and STAT3 and/or through suppression of beclin 1 synthesis^50–52^.

The STAT3-driven pathway of increased lysosomal capacity was observed in different cell types and is likely to be a general response to lysosomal stress-induced by protease insufficiency/substrate accumulation. It may however be particularly relevant in the kidney whose PTC are tasked with reabsorption and processing of a continual macromolecule load present in the glomerular ultrafiltrate. Faced with an increased protein load as occurs in proteinuria, PTC likely need to increase lysosomal capacity. Indeed, in focal segmental glomerulosclerosis (FSGS), a model of glomerular injury, proteinuria led not only to increased lysosome protein content but also to an apparently adaptive increase in some lysosomal proteases^53^. A mechanistic basis for this was not elucidated but based on our results it seems likely that proteinuria leads to lysosomal stress, activation of STAT3 in PTC and that this is the basis of increased biosynthesis of lysosomal hydrolases. Activation of STAT3 as a result of lysosomal stress may explain some of the inflammatory features of proteinuric kidney disease. Linked to this, other studies have shown that protein overloaded proximal tubular cells express several vasoactive and inflammatory mediators^54^ and that the Jak/STAT pathway was activated following protein overload of PTC in vitro^55^. With a full complement of lysosomal protease genes available, resolution of lysosomal overload would be expected to extinguish the oxidative stress that drives STAT3 activation and consequently, return hydrolase transcription to normal levels. However, when a critical gene such as AEP is missing and accumulation of target substrates continues, STAT3 activation becomes chronic likely explaining the onset of the severe proliferative kidney disease in AEP-null mice^26^.

Oxidative stress is a common feature of lysosomal storage diseases (LSD) but how these diverse genetic deficiencies lead to oxidative stress is not fully understood^43^ and this is also the case in AEP-deficient cells. However, it is known that loss or mutation of another lysosomal protease, CtsD can lead to congenital neuronal ceroid-lipofuscinosis characterised by accumulation of lipofuscin laden vacuoles and oxidative damage to brain pericytes and other cells^16^. Other studies show that intracranial administration of the pan-lysosomal cysteine protease inhibitor, leupeptin also led to ceroid–lipofuscin accumulation in rat brain^56^. Lipofuscin is a complex undegraded material, rich in protein, lipid, carbohydrates and transition metals, that is associated with the generation of reactive oxygen species^57^. Thus while the details of how oxidative stress-induced by protease/substrate imbalance remain to be clarified, there is clear evidence that lysosomal proteases are non-redundantly required for normal lysosomal homoeostasis and that their loss leads to various forms of lysosomal stress and downstream pathology.

The lysosome-mediated pathway of cell death has been well characterised^58^ but has only recently been shown to be physiologically important. Watson and colleagues have provided striking evidence that post-lactational involution of the mammalian mammary gland is achieved by lysosome-mediated programmed cell death (LM-PCD), dependent on STAT3 activation^7,8^. STAT3 increased expression of CtsB and CtsL and increased uptake of milk fat globules which, upon hydrolysis, released lysosome-permeabilising unsaturated fatty acids which were essential for the induction of LM-PCD. We suggest that the STAT3-driven pathway of lysosomal hydrolase expression may primarily be one for lysosomal adaptation to greater substrate load as shown here. However, as shown by studies in the involuting mammary gland, when combined with perturbations to the lysosomal membrane it can also be used to drive LM-PCD.

In summary, our results shed light on how cells can adjust their lysosomal hydrolytic capacity under conditions of nutritional sufficiency rather than insufficiency which leads to TFEB activation. Communication between the lysosomal lumen and the cytosol appears to depend on lysosomal stress signals rather than lysosomal amino acid levels. Finally, there is great interest in manipulation of the lysosomal system, for example through TFEB activation, to enhance clearance of protein aggregates that can lead to pathology for example in neurodegenerative disease. Our results may suggest additional strategies involving the STAT3 signalling axis and potentially AEP inhibition, which as shown here can lead to extensive adaptational changes that overall, boost lysosome performance.

## Methods

### Antibodies

All antibodies used in this manuscript were used a 1:1000 dilution when used for immunoblotting and 1:200 when used for immunofluorescence. Antibodies against P-STAT3 (D3A7), STAT3 (124H6), P-Jak2 (D2E12), Jak2 (3771A), P-4EBP1 (236B4), LC3 (D3U4C) and GAPDH (14C10) were obtained from Cell Signaling. Antibodies against Laminin (ab11575), CtsD (ab75852), CtsB (ab92955), Lamp1 (ab24170) and Fibronectin (ab199056) were purchased from AbCam. Anti-Ki67 (B56) obtained from BD Bioscience, anti-Gns (13044-1-AP) from ProteinTech, anti-TFEB (A303-673A) from Bethyl, anti-YM1 (60130) from Stem Cell Technologies, anti-EGFR (sc-03) from SantaCruz, anti-OSMRβ (3458-100) from R&D System and anti-SOD1 (3458-100) from BioVision.

### Mice and cell culture

WT and AEP^−/−^ mice on a C57BL/6 background, generated as previously reported^59^, were bred and maintained under pathogen-free conditions under UK Home Office Project License PPL60/3851 and with project approval from the University of Dundee Ethical Review Committee. WT and AEP-null primary murine embryonic fibroblast (MEF) were generated from WT or AEP^−/−^ mice as previously described^60^ and expanded using complete Dulbecco’s modified Eagle medium (DMEM) supplemented with 100 U/ml penicillin, 100 μg/ml streptomycin, 2 mM l-glutamine, 1% pyruvate, 1% non-essential amino acids and 10% FBS. For protein overload experiments, wildtype MEF were incubated with bovine serum albumin (30 mg/ml) on coverslips for immunofluorescence experiments or in 6 well plates to measure lysosomal proteolytic activities. HKC-8, a human-derived proximal tubule cell line^33^, were cultured in 1:1 Dulbecco’s modified Eagle’s—F12 medium supplemented with 100 U/ml penicillin, 100 μg/ml streptomycin, 2 mM l-glutamine and 5% FBS.

### Isolation of human primary proximal (PTC) and distal tubular cells (DTC)

Human kidneys cells were isolated from adult kidneys after surgical resection in accordance with the Research Ethics Committee guidelines and approval granted by the NRES Committee East Midlands-Derby (REC reference 13/EM/0311) as previously described^61^. Informed patient consent was obtained as part of this approval. Kidney cortex was minced and digested with 1 mg/ml collagenase IV for 60 min at 37 °C and passed through a 40 µm cell strainer. After centrifugation at 300×*g*, the pellet was resuspended in RPMI and loaded in a two layer Percoll gradient (50%, 24.6%). Centrifugation at 1000×*g* for 25 min at 4 °C produced three separate layers: a top layer containing DTC and a middle layer containing PTC were retrieved and separately washed twice in RPMI at 300×*g*. DTC were seeded and maintained in DMEM/F-12, GlutaMAX™ supplemented with 100 U/ml penicillin, 100 µg/ml streptomycin, 2 mM l-glutamine, 10% FBS and maintained at 37 °C in an atmosphere of 5% CO_2_. PTC were seeded and maintained in REGM BulletKit media from Lonza supplemented with 100 U/ml penicillin, 100 µg/ml streptomycin. Cells were used between passage 2 and 3. Phenotypic PTC and DTC confirmation was obtained by electron microscopy.

### Transmission electron microscopy

The kidney cortex was cut into small (1 mm thick) pieces, and immediately fixed in Karnovsky’s fixative (2.5% glutaraldehyde, 4% paraformaldehyde in 100 mM cacodylate buffer pH 7.2). After washing twice in cacodylate buffer and post-fixing in 1% OsO_4_ in cacodylate buffer for 60 min, the samples were dehydrated in alcohol, transferred to 100% propylene oxide and finally incubated overnight on a rotator in 50% propylene oxide: 50% Durcupan resin (Sigma). After transfer to 100% Durcupan resin and polymerisation at 60 °C overnight, sections (70–100 nm thick) were cut using an ultramicrotome (Leica Ultracut UCT) and stained with 3% uranyl acetate followed by Reynolds lead citrate (1.33 g lead nitrate, 1.76 g sodium citrate, 8 ml 1 M NaOH in 50 ml water). Grids were imaged on a JEOL 1200 TEX TEM and images were collected on Digital Imaging Plates and scanned on a Ditabis Micron scanner (Pforzheim, Germany).

### Enzymatic activity assays

Cell lysates were prepared in 0.2 M Na citrate buffer pH 4.0 containing 1% Triton X-100. To measure AEP and CtsB/L activities 20 μg of total protein were incubated in 200 μl assay buffer (0.2 M Na citrate buffer pH 4.0, 1 mM DTT) containing 10 μM AEP substrate [Z-Ala-Ala-Asn-7-amino-4-methyl coumarin (AMC)] or 10 μM CtsB/L substrate [Z-Phe-Arg-AMC], respectively. AMC release was quantified at 460 nm in a fluorescence plate reader (Fluorostar Optima, BMG Labtech), and activities calculated as arbitrary units. CtsD/E activities were measured under the same conditions but using 50 μg of cell lysate, 10 μM CtsD/E substrate [MCA-Gly-Lys-Pro-Ile-Leu-Phe-Arg-Leu-Lys(Dnp)-D-Arg-NH2] and MCA release measured at 390 nm. Activities were calculated as arbitrary units.

Superoxide dismutase [Cu-Zn] (SOD1) activity was determined using 20 μg of total cell lysate (TBS plus 1% Triton X-100) from WT or AEP^−/−^ MEF treated or untreated with different inhibitors using the Superoxide Dismutase Assay Kit following the manufacturer’s instructions (ScienCell Research Laboratories).

### Immunofluorescence microscopy

Kidneys were removed immediately from culled WT and AEP^−/−^ mice and fixed in 10% buffered formalin and embedded in paraffin blocks. Sections (5 μm thick) were cut, deparaffinised using Histo-Clear and subjected to antigen retrieval using pressure cooking in Na citrate buffer, pH 6.0. Sections were permeabilized with 1× TBS supplemented with 0.025 % Triton x-100 and blocked with 10% FBS and 1% BSA in TBS prior to overnight staining at 4 °C with the following primary antibodies prepared in TBS, 1% BSA: rabbit anti-P-STAT3, mouse anti-Ki67, rabbit anti-laminin, rabbit anti-CtsD, rabbit anti-Gns, rabbit-anti-TFEB, rabbit anti-P-4EBP1 (dilution 1:200). After washing in TBS 0.025% Triton X-100 twice, the slides were incubated for 1 h with the appropriate Alexa-488- or Alexa 594-conjugated secondary antibody (Invitrogen, dilution 1:500) in TBS with 1% BSA. The slides were finally washed three times in TBS and mounted under coverslips using Vectashield mounting medium containing DAPI (Vector Laboratories) and viewed using an LSM 700 confocal microscope (Carl Zeiss).

WT and AEP^−/−^ MEF (5 × 10^4^ cells/coverslip) were fixed with 4% paraformaldehyde in PBS at room temperature for 20 min or with 100% methanol at −20 °C for 10 min. The cells were washed three times with PBS, permeabilized with 0.2% Triton X-100 in PBS for 20 min at room temperature, washed 3× with PBS and then incubated for 20 min at room temperature in blocking buffer (1% BSA in TBS). The cells were stained using a 1:200 dilution of primary antibody in blocking buffer for 45 min at room temperature (rabbit anti-TFEB, rabbit anti-LC3 or rabbit anti-P-STAT3), washed three times with PBS and stained with a 1:500 dilution of the Alexa-488-conjugated secondary antibody (Invitrogen) for another 45 min at room temperature. Coverslips were washed three times with PBS 1× and mounted using Vectashield mounting medium containing DAPI (Vector Laboratories) and viewed using an LSM 700 confocal microscope (Carl Zeiss).

### STAT3-YFP and TFEB-GFP cellular distribution in HKC-8 cells

The TFEB-GFP construct was purchased from Acris Antibodies. STAT3-YFP was cloned into pcDNA5. HKC-8 cells (5 × 10^4^ cells/coverslip) were transfected with STAT3-YFP or TFEB-GFP using polyethylenimine (PEI)^62^ and 24 h later cells were treated as indicated in the figure legends. After fixation with 4% paraformaldehyde in PBS at room temperature for 20 min the coverslips were washed three times with PBS 1× and mounted using Vectashield mounting medium containing DAPI (Vector Laboratories) and viewed using an LSM 700 confocal microscope (Carl Zeiss).

### TFEB knockout generation in HKC-8 cells

Two different TFEB knockouts were generated by targeting TFEB exon 1 or exon 2. The sense guides (exon 1: GCATGCAGCTCATGCGGGAGC; exon 2: GTGCCCCAGCAGCATCCCCAG) were cloned into the pBABED puro U6 and the antisense guides were cloned into the Cas9 D10A vector pX355 (exon 1: GCAGCCCGATGCGTGACGCCA; exon 2: GGCTTCGGGGAACCTTGGGC).

HKC-8 cells (10^6^ cells/10 cm dish) were transfected with the sense and antisense guide plasmids targeting exon 1 or exon 2 of the TFEB gene using PEI. Twenty-four hours later the media was replaced with media supplemented with 2 μg/ml puromycin. After 48 h of puromycin selection transfected cells were expanded and individual clones were isolated and tested for TFEB expression by western blotting.

### Jak2 knockdown in HKC-8 cells

A set of four Jak2-siRNAs were purchased from Dharmacon and tested individually to determine levels of knockdown achieved. The siRNAs providing the highest level of Jak2 knockdown (siRNA #6: GAAUUGUAACUGUCCAUAA and siRNA #7: GAAUUUAUGCGAAUGAUUG) were subsequently used in all the experiments.

HKC-8 cells (10^4^ cells per well) in a 96 well plate were transfected with Jak2-siRNAs or non-targeting siRNA (UGGUUUACAUGUCGACUAA) (Dharmacon) using DharmaFect 1 transfection reagent (Dharmacon) following the manufacturer’s instructions. Forty-eight hours later cells were treated as indicated and samples prepared for SDS-PAGE analysis.

### STAT3 knockdown and rescue in HKC-8 cells

A set of four STAT3-siRNAs were purchased from Dharmacon and tested individually to determine levels of knockdown achieved. The siRNAs providing the highest level of STAT3 knockdown (siRNA #8: CAACAUGUCAUUUGCUGAA and siRNA #10: CAACAGAUUGCCUGCAUUG) were subsequently used in all the experiments. HKC-8 cells (10^4^ cells per well) in a 96 well plate were transfected with STAT3-siRNAs or non-targeting siRNA (UGGUUUACAUGUCGACUAA) (Dharmacon) using DharmaFect 1 transfection reagent (Dharmacon) following the manufacturer’s instructions. Forty-eight hours later cells were treated as indicated and samples prepared for SDS-PAGE analysis

To generate a form of STAT3 refractory to siRNA knockdown, we fused it to GFP to distinguish it from endogenous STAT3 (mSTAT3-GFP) and introduced mutations into the target sequence for siRNA #8 (see above) using the following primers 5′-GCAGCTGAACAACATGTCATTTGCTGAAATCATCATG-3′ and 5′-CATGATGATTTCAGCAAATGACATGTTGTTCAGCTGC-3′. HKC-8 cells were transiently transfected using PEI and GFP-positive cells were enriched by FACS using an Influx cell sorter (Becton Dickinson). Cells were distinguished from debris based on FSC-H vs SSD-H and single cells on the basis of FSC-A vs FSC-W measurements. GFP-positive cells were identified based on background fluorescence levels in the parental cell line negative control. Then, sorted cells were expanded for 48 h in the presence of G418 to maintain plasmid expression prior to siRNA (#8 or non-targeting) transfection. Knockdown of endogenous STAT3 versus mSTAT3-GFP was assessed by western blotting and STAT3 activation by MVO-treatment was performed and analysed as previously described

### STAT3 ChIP-PCR

Anti-STAT3 ChIP was carried out as previously reported^63^. Briefly, WT and AEP^−/−^ MEFs were grown to 70% confluency, crosslinked with 1% formaldehyde for 10 min at RT. Excess formaldehyde was blocked with 0.125 M glycine and the cells washed with ice-cold PBS several times after which the cells were scraped off in 2 ml of PBS containing protease inhibitors and collected by centrifugation at 500×*g* for 10 min at 4 °C. Pellets were resuspended in lysis buffer (1% SDS, 10 mM EDTA, 50 mM Tris–HCl (pH 8.1) and protease inhibitors) and sonicated three times for 5 min in a BioRuptor RTH (30 s on/30 s off). Samples were then diluted 1:10 in ChIP dilution buffer (1% Triton X-100, 2 mM EDTA, 150 mM NaCl, 20 mM Tris–HCl (pH 8.1)) and anti-STAT3 antibody was added to the samples (Cell Signaling, 124H6) and incubated overnight at 4 °C. Finally immunoprecipitated chromatin was purified using the iPure Kit (Diagenode) following the manufacturer’s instructions.

The samples were then tested for the presence of the promoter regions of genes of interest by PCR using the following primers:

STAT3 (forward: GGTGACACCTGGGGACCGCCTAAG; reverse: AAAAACGCCTCTAGGAGAGAAGGCG),

AEP (forward: ATGAGTTGGCCCTGTTTTGGGTCC; reverse: TCTGCCTTGGCCTATGATCACAAC),

CtsL (forward: AAAACAGTCTCCATCGTTCTCAAC; reverse: GGCCTGCTGTGGAATTTCCACCTC),

CtsD (forward: AGAGTGAAGTAGTTAAGACCCAAG; reverse: TACCAGGATGTGGTCTTTGTCTGA),

Gns (forward: GGAAGGTGCGTGAGCACGCAAAAC; reverse: GGACAGAGCACCGGAGATGAAGAC),

Man2b2 (forward: GATAGAGAGGGATCTGAGCTTGCT; reverse: CAGAATCAAATCTGGGGTCTTGGA),

Naga (forward: GTTAAGAGCAAGGTTTCAGGTGGA; reverse: GTTCTGCGCGTTCCCGAGGCGCGC),

Plbd2 (forward: TGCAGAGGACTTGAGTTCGAATTC; reverse: CTCAGCATCCTTCCCTCCCAAGGA).

The sequence of the primers used as negative controls was: (forward: ATGGTTGCCACTGGGGATCT; reverse: TGCCAAAGCCTAGGGGAAGA)

Electrophoretically separated PCR products were quantified with ImageJ and normalised to input samples.

### Generation of ^13^C_6_-Lysine SILAM mouse colony

An in-house breeding colony of C57BL/6 ^13^C_6_-Lysine ‘SILAM’ labelled mice was generated using specialised ^13^C_6_-Lysine mouse diet (Silantes, Martinsried, Germany) following the breeding programme previously described by Krüger et al.^29^ Labelling efficiency was monitored for brain, liver and skin tissues from 19 weeks old F0 (fed ^13^C_6_-Lysine mouse diet for 16 weeks), 8-week-old F1 and 8-week-old F2 animals. Labelling efficiencies for all confidently (1% false discovery rate) assigned peptides were calculated as: heavy_peptide_area /(heavy_peptide_area + light_peptide_area). For all tested samples, ~90% of the peptides were > 90% ^13^C_6_-Lysine-labelled at the F2 generation. Tissues used for this study were acquired from F4 C57BL/6 ^13^C_6_-Lysine SILAM females.

### Kidney lysosome SILAC-based mass spectrometry

Kidney lysosomes from l-lysine-^13^C_6_ labelled WT mice and unlabelled WT or AEP^−/−^ mice were prepared as previously described^26^. 60 μg of unlabelled WT or AEP^−/−^ kidney lysosomal proteins were combined in a 1:1 ratio with 60 μg of l-lysine-^13^C_6_ labelled WT kidney lysosomal proteins. Protein samples were prepared for LC-MS/MS using the FASP protocol as previously described^64^. Briefly, the protein mixture was denatured at 95 °C for 5 min in SDS-lysis buffer (4% (w/v) SDS, 100 mM Tris–HCl pH 7.6, 0.1 mM DTT) and the samples were mixed with 200 μl of 8 M urea in 0.1 Tris–HCl pH 8.5 (UA buffer) in a Microcon YM-10 filter unit and centrifuged at 17,000×*g* for 40 min. Samples were washed again with another 200 μl of UA buffer and then treated with 100 μl of 0.05 M iodoacetamide in UA buffer for 20 min at room temperature. After three further washes with 100 μl UA buffer and three times with 0.05 M NH_4_CO_3_ (ABC buffer) centrifuging each time at 14,000×*g* for 15 min and discarding the flow through, the protein mixture was digested overnight at 37 °C in the YM-10 filter unit using 40 μl of 0.4 μg/μl trypsin in ABC buffer. The samples containing the tryptic peptides were collected by centrifugation at 14,000×*g* for 40 min and 50 μl of 0.5 M NaCl was added to the YM-10 filters followed by centrifugation for another 20 min at 14,000×*g*. Tryptic peptides were finally desalted using a C18-SD cartridge, treated with 0.1% trifluoroacetic acid and eluted in 70% acetonitrile.

### MEF SILAC-based mass spectrometry

MEF were cultured for at least 10 days in RPMI without arginine and lysine (Biosera) containing 10% dialysed FCS (Hyclone), 100 U/ml penicillin, 100 μg/ml streptomycin, 2 mM l-glutamine, 1% pyruvate, 1% non-essential amino acids and supplemented with 84 mg/l of l-arginine (Sigma) and 40 mg/l of l-lysine (Sigma) for the ‘light’ medium or 106 mg/ml of l-arginine-^13^C_6_-^15^N_4_ and 50 mg/ml of l-lysine-^13^C_6_ for the ‘heavy’ medium. Equal numbers of cells were mixed and cytoplasmic, membrane and nuclear fractions were obtained using a subcellular protein fractionation kit for cultured cells (Thermo) according to the manufacturer’s instructions. 100 μg of protein was size-fractionated by SDS-PAGE using 4–12% NuPAGE gels (Invitrogen) in MES running buffer, stained using SimplyBlue Safe Stain (Invitrogen) and destained using miliQ water. The gels were cut into 15 × 0.5 cm slices using a clean scalpel and cut into 1 mm cubes. After washing with 50% acetonitrile/water, 0.1 M NH_4_CO_3_ and 50% acetonitrile in 50 mM NH_4_CO_3_ (0.5 ml) the samples were treated with 75 μl of 10 mM DTT in 0.1 M NH_4_CO_3_ and incubated for 20 min at 37 °C. The liquid was removed and proteins in the gel pieces were carboxymethylated with 50 mM iodoacetamide in 0.1 M NH_4_CO_3_ for 20 min at room temperature in the dark. The gel pieces were washed with 50% acetonitrile in 50 mM NH_4_CO_3_, shrunk using 0.3 ml acetonitrile per piece for 15 min and after removal of the supernatant, dried using a Speed-Vac lyophiliser. The protein content of each piece was digested with 30 μl 5 μg/ml of trypsin in 25 mM triethylammonium bicarbonate at 30 °C overnight. An equal volume of acetonitrile was added and the mixture was shaken at 30 °C for 15 min. The supernatants, containing the tryptic peptide mixture from each sample, were frozen in liquid nitrogen and dried using a Speed-Vac. Meanwhile, the gel pieces were treated with 100 μl of 50% acetonitrile, 2.5% formic acid in water and shaken for 10 min at 30 °C. This second supernatant was combined with the first, frozen down in liquid nitrogen and then dried in a Speed-Vac.

### Mass spectrometry

A nanoflow liquid chromatograph (Ultimate 3000 RSLCnano system, Thermo) coupled to a LTQ Orbitrap Velos Pro mass spectrometer (Thermo) was used to analyse the protein digests. Samples, usually 10 μl, were loaded onto a C18 trap column and washed with 0.1% formic acid. The peptides were resolved using a gradient (130 min, 0.3 μl/min) of buffer A (0.1% formic acid) and buffer B (80% acetonitrile in 0.08% formic acid): 2% buffer B for 4 min followed by 2–40% buffer B for 64 min, 40–98% buffer B for 2 min, 98% buffer B for 15 min, 98–2% buffer B for 1 min and 2% buffer B for 44 min. Peptides, initially trapped on an Acclaim PepMap 100 C18 column (100 μm × 2 cm, Thermo) were then separated on an Easy-Spray PepMap RSLC C18 column (75 μm × 50 cm, Thermo), and finally transferred to the LTQ Orbitrap Velos Pro via an Easy-Spray source set at 50 °C and a source voltage of 1.9 kV. For the identification of peptides, a top 15 method (FT-MS plus 15 IT-MS/MS, 100 min acquisition) consisting of full scans and mass range (*m*/*z*) between 335 and 1800 was used. The Orbitrap was operated in a profile mode, resolution of 60,000 with a lock mass set at 445.120024 and a max fill time of 500 ms. LTQ was operated in a centroid mode with isolation width = 2.00 (*m*/*z*), normalised collision energy = 35.0, activation time = 10.0 ms and max fill time of 100 ms.

### Mass spectrometry data analysis

LTQ Orbitrap Velos Pro.RAW files were analysed, and peptides and proteins quantified using MaxQuant^65^, using the built-in search engine Andromeda^66^. All settings were set as default, except for the minimal peptide length of 5, and Andromeda search engine was configured for the UniProt *Mus musculus* protein database (release date: 2013_10). Peptide and protein ratios only quantified in at least two out of the three replicates were considered, and the *p*-values were determined by Student´s *t* test and corrected for multiple testing using the Benjamini–Hochberg procedure^65^.

### Sample preparation and immunoblotting

Tissue samples were prepared using a 7-ml dounce tissue grinder (Wheaton, USA) in complete RIPA buffer (RIPA buffer (Thermo) supplemented with 1 mM sodium orthovanadate, 10 mM sodium fluoride and 1 protease inhibitor tablet (Roche)). WT and AEP^−/−^ MEFs (2 × 10^5^ cells) treated or untreated with the different inhibitors were washed once with ice-cold PBS and lysed using complete RIPA buffer. Tissue and cell lysates were centrifuged at 20,000×*g* for 10 min and equal amounts of protein loaded on 4–12% NuPAGE gels (Invitrogen) and then transferred onto nitrocellulose membranes (Amersham). Membranes were probed with the following antibodies: rabbit anti-P-STAT3, mouse anti-STAT3, rabbit anti-GAPDH and rabbit anti-LC3 (Cell Signaling), rabbit anti-Ym1 (StemCell Technologies), rabbit anti-EGFR (SantaCruz), rabbit anti-Lamp1, rabbit anti-CtsD, rabbit anti-Fn1 and anti-CtsB (abCam), rabbit anti-TFEB (Bethyl) and rabbit anti-Gns (ProteinTech) and goat anti-OSMRβ (R&D Systems). Sheep anti-AEP was obtained as described previously^32^ and sheep anti-CtsL and CtsH were generous gifts from Drs Tina Zavašnik-Bergant and Janko Kos (Jožef Stefan Institute, Ljubljana, Slovenia)

### In vivo cathepsin L activity assay

WT and AEP^−/−^ MEF (5 × 10^4^ cells) were grown on coverslips and incubated with Magic Red cathepsin L (Immunochemistry Technologies) for 60 min at 37 °C following the manufacturer’s instructions. The cells were then fixed with 4% paraformaldehyde in PBS, mounted using Vectashield mounting medium containing DAPI (Vector Laboratories) and images were obtained using a LSM 700 confocal microscope (Carl Zeiss).

### Lysosomal degradation of DQ-BSA

WT 3T3 cells (5 × 10^4^ cells) were grown on coverslips and treated with MVO 50 μM for 16 h. Then, cells were incubated with 10 μg/ml of DQ-BSA (Invitrogen) or 50 μg/ml of FiTC-BSA for 2 h at 37 °C, washed twice with PBS, then fixed with 4% paraformaldehyde in PBS for 20 min at room temperature, mounted using Vectashield mounting medium with DAPI (Vector Laboratories) and images were obtained using an LSM 700 confocal microscope (Carl Zeiss).

### Onconstatin M receptor β (OSMRβ) and epithelial growth factor receptor (EGFR) degradation assay

Total cell extracts from WT cells treated or not with 50 μM MVO26630 and AEP^−/−^ MEF (2 × 10^5^ cells), incubated as indicated with oncostatin M 25 ng/ml (R&D Systems) or epithelial growth factor 100 ng/ml and cycloheximide (10 μg/ml), were prepared in complete RIPA buffer and equal amounts of protein were separated by SDS-PAGE and subjected to direct Western blotting using anti-OSMRβ or anti-EGFR as described above.

### In-cell reactive oxygen species determination

WT and AEP^−/−^ MEF grown on FluoroDish dishes (World Precision Instruments Inc.) treated as indicated with the different inhibitors were incubated with 100 nM LysoTracker Green DND26 (Invitrogen) for 2 h at 37 °C and then with 2.5 μM CellROX Orange (Invitrogen) for 30 min at 37 °C. The cells were washed twice with PBS, fresh complete DMEM was added to the dishes and live cell imaging carried out at 37 °C and 5% CO_2_ using a LSM 700 confocal microscope (Carl Zeiss). CellROX Orange intensity overlapping with LysoTracker Green DND26 positive structures was quantified using the Volocity 3D Image Analysis Software (PerkinElmer). The mean intensity of CellROX Orange per LysoTracker Green DND26 positive structure, normalised for lysosomal size (µm^2^) and per cell was calculated and the standard error calculated using 15 cells per experimental condition.

### Semi-quantitative reverse transcription-polymerase chain reaction (RT-PCR)

Total RNA from WT MEF, treated or untreated with 50 μM MVO26630 overnight, and AEP^−/−^ MEFs, WT and STAT3c 3T3s was purified using RNeasy mini kit (Qiagen) following the manufacturer’s instructions. Total RNA concentration was determined using a NanoDrop 1000 spectrophotometer (Thermo) and 1 μg of total RNA per sample was used to synthesise cDNA using the qScript Flex cDNA kit (Quanta Biosciences) according to the manufacturer’s conditions. Finally, the mRNA expression levels of different lysosomal proteases were determined by PCR, quantified using ImageJ (imagej. nih.gov) and normalised using β-actin or Tubulin 5 as loading control. The following primers were used: β-actin: forward (ATCATGTTTGAGACCTTCAACA), reverse (CATCTCCTGCTCGAAGTCTA); Tubb5: forward (TGTACTATAATGAAGCCACAGGTGG), reverse (CAGTAGGTCTCATCCGTGTTCTCAA); AEP: forward (ATGACCTGGAGAGTGGCTG), reverse (CGTTGATGTCGTCGGGCA); CtsB: forward (GGGTACTTAGGAGTGCACGG), reverse (CCAAATGCCCAACAAGAGCC); CtsD: forward (TCAGGAAGCCTCTCTGGGTA), reverse (CTGCAGCTCCTTCACCTCTT); CtsH: forward (ATGACGAGGCTGCAATGGTT), reverse (TCCCTTGGTCTGCCAATCAG); CtsL: forward (ATGGCACGAATGAGGAAGAG), reverse (GAAAAAGCCTCCCCTTCTTG) and CtsZ: forward (CAGCGGATCTCCCCAAGAAT), reverse (GTCCTTGGCCTGGTAGTTGT).

Total RNA from HKC-8 WT or TFEB KOs was purified using RNeasy mini kit (Qiagen) following the manufacturer’s instructions. Total RNA concentration was determined using a NanoDrop 1000 spectrophotometer (Thermo) and 1 μg of total RNA per sample was used to synthesise cDNA using the qScript Flex cDNA kit (Quanta Biosciences) according to the manufacturer’s conditions. Finally, the mRNA expression levels of different lysosomal proteases were determined by PCR, quantified using ImageJ (imagej. nih.gov) and normalised using β-actin as loading control. The following primers were used: β-actin: forward (GGGGTGTTGAAGGTCTCAAA), reverse (GGCATCCTCACCCTGAAGTA); CtsB: forward (CGCTTTCCATTCCTGGGTCTCTG), reverse (ACAGGCCATGTGAGCCACCG); CtsD: forward (CCCACACACACCCACACACTCG), reverse (CCAGGGAGGGGAAAACCACAGA).

### HKC-8 WT AND TFEB KOs Starvation

Total cell extracts from HKC-8 WT and TFEB KO cells starved or not for 4 h in HBSS at 37 °C and 10% CO_2_ were prepared in complete RIPA buffer and equal amounts of protein were separated by SDS-PAGE and subjected to direct Western blotting using anti-LC3 or anti-CtsB as described above.

### GO analysis

ClueGo^66^ within the Cytoscape software^67^ was used to find statistically over-represented gene ontology (GO) categories. The identified proteins from the kidney lysosomal SILAC-based mass spectrometry analysis were used as a test dataset and a reference set of GO annotations for the mouse proteome was used in order to carry out the GO enrichment analysis. Analyses were done using a right-sided hypergeometric test and all GO terms corresponding to cellular location or molecular function with *p* < 0.005 were selected.

## Supplementary information

Supplementary Information

Peer Review File

Supplementary Data 1

## Data Availability

The authors declare that all primary data are available in main or Supplementary figures. The mass spectrometry proteomics data have been deposited to the ProteomeXchange Consortium via the PRIDE partner repository with the dataset identifier PXD011473. All other data that support the findings of this study are available from the corresponding authors upon reasonable request
