# Supplementary material for: Lysosomal protease deficiency or substrate overload induces an oxidative-stress mediated STAT3-dependent pathway of lysosomal homeostasis
Source: Nat Commun. 2018 Dec 17;9:5343. doi: 10.1038/s41467-018-07741-6 (PMC6297226; doi:10.1038/s41467-018-07741-6)
Supplement: Supplementary file 1 — Supplementary Information [file 41467_2018_7741_MOESM1_ESM.pdf]

Supplementary Information

J. Martinez-Fabregas et al

Lysosomal protease deficiency or substrate overload induces an oxidative-stress mediated STAT3-dependent pathway of lysosomal homeostasis

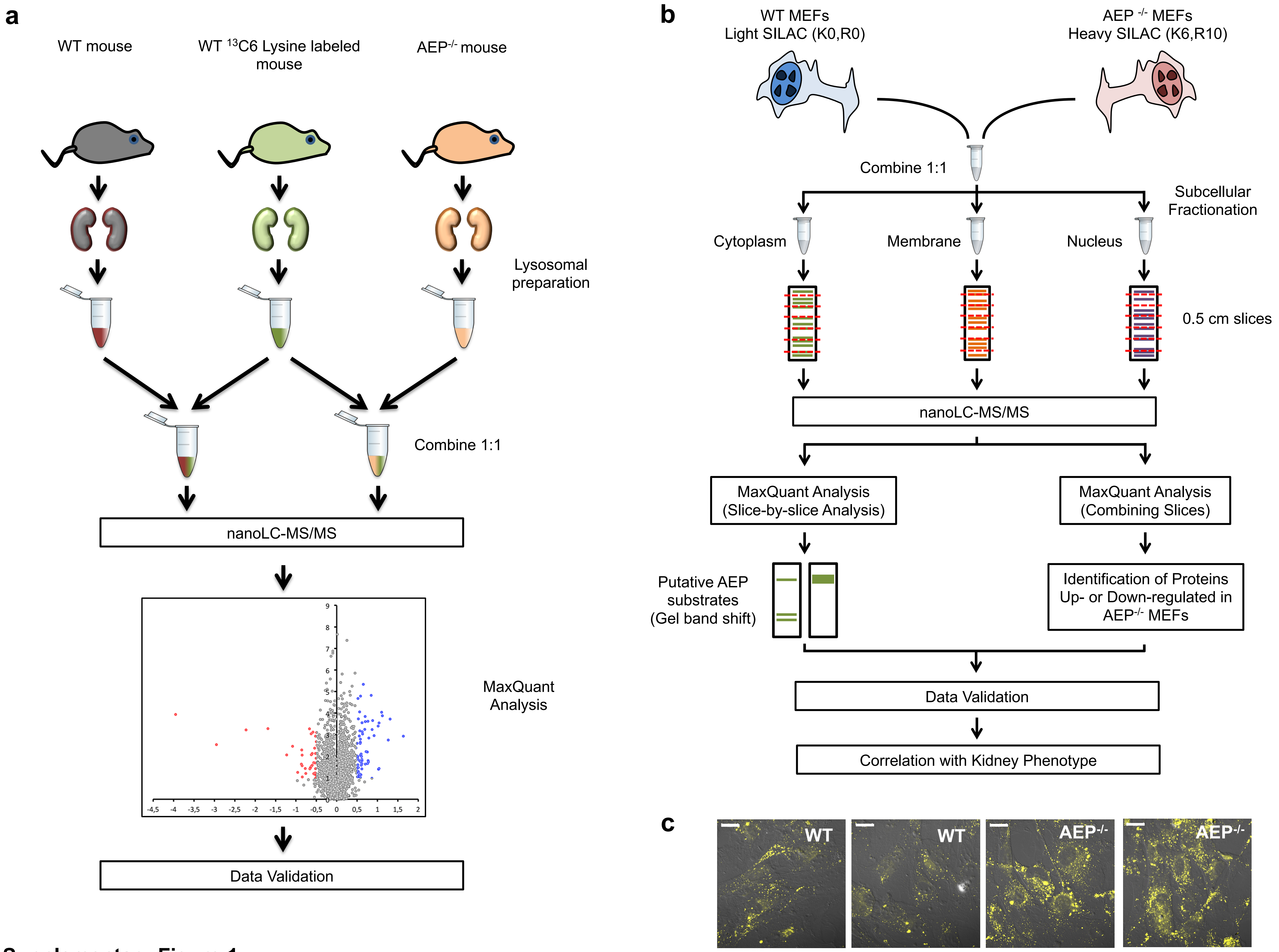

Supplementary Figure 1

a

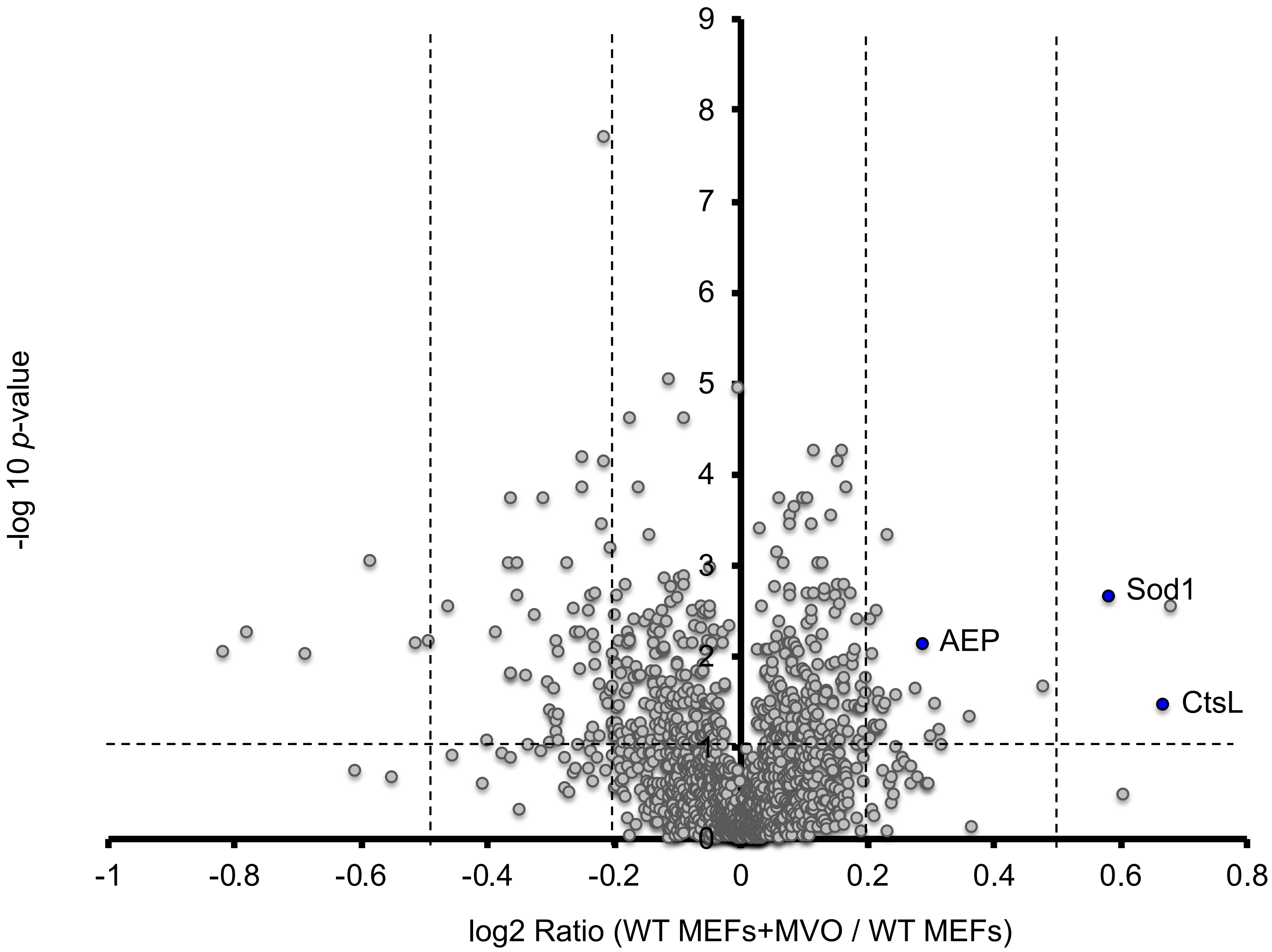

b

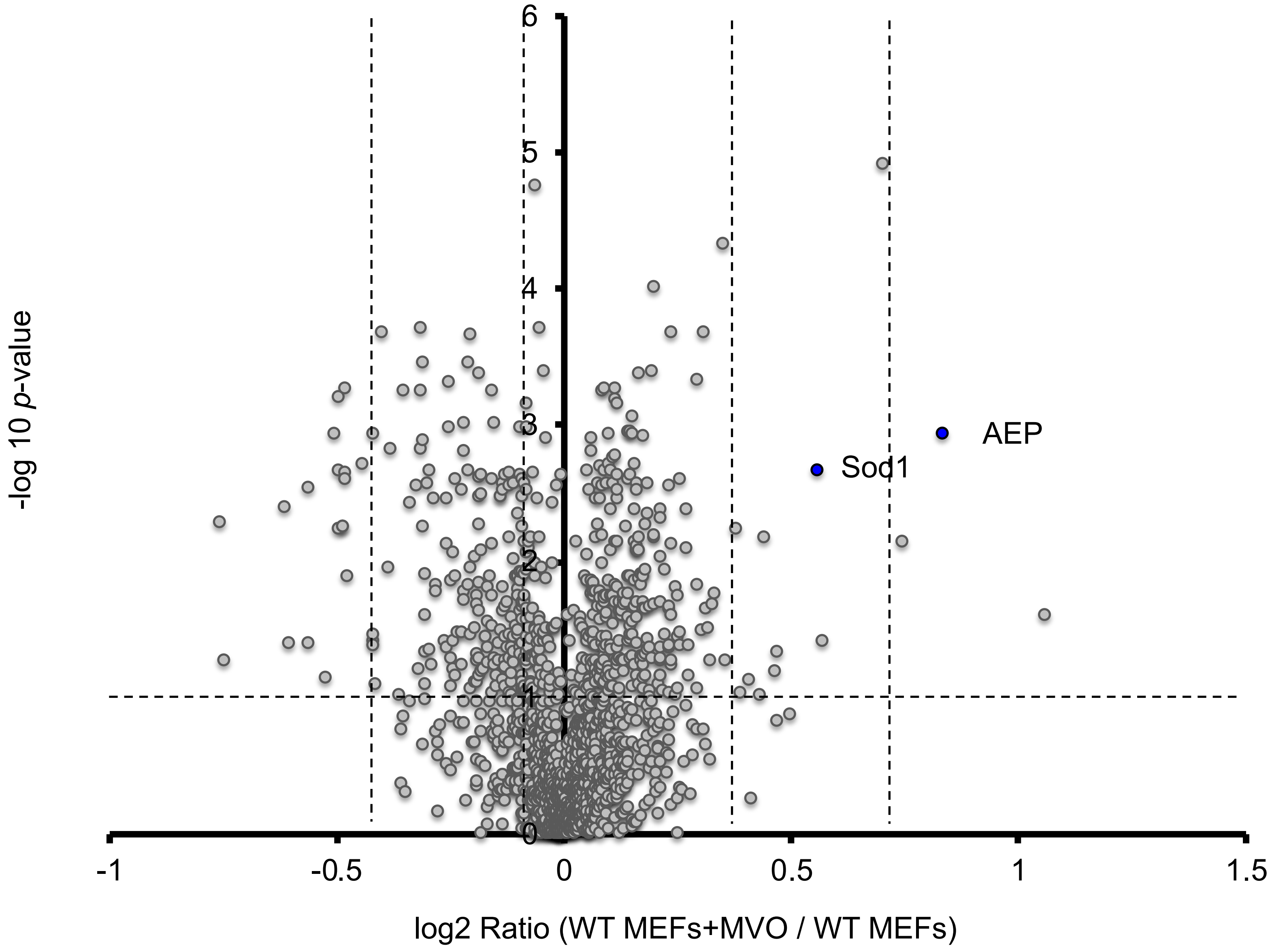

Supplementary Figure 2

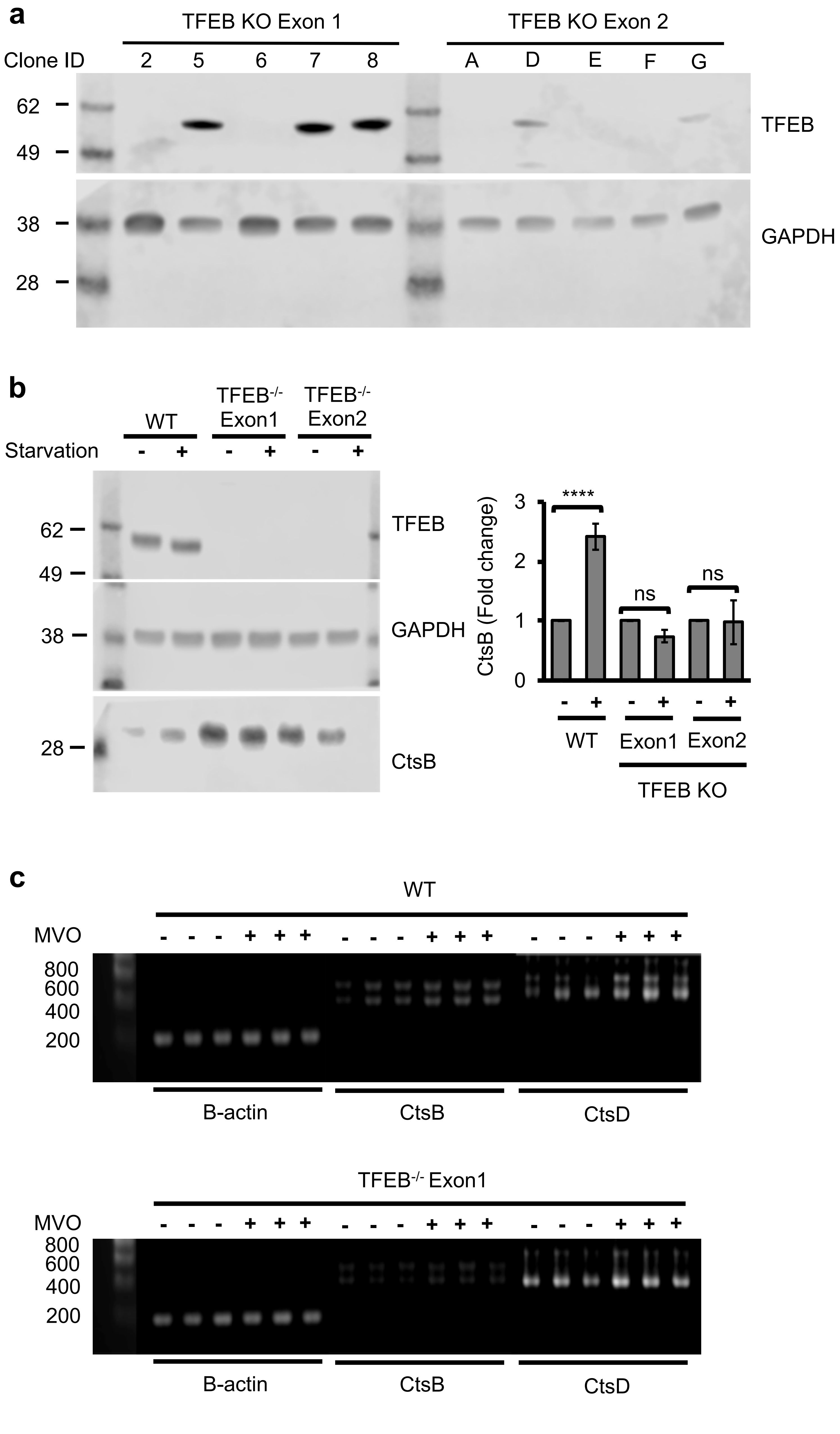

Supplementary Figure 3.

a

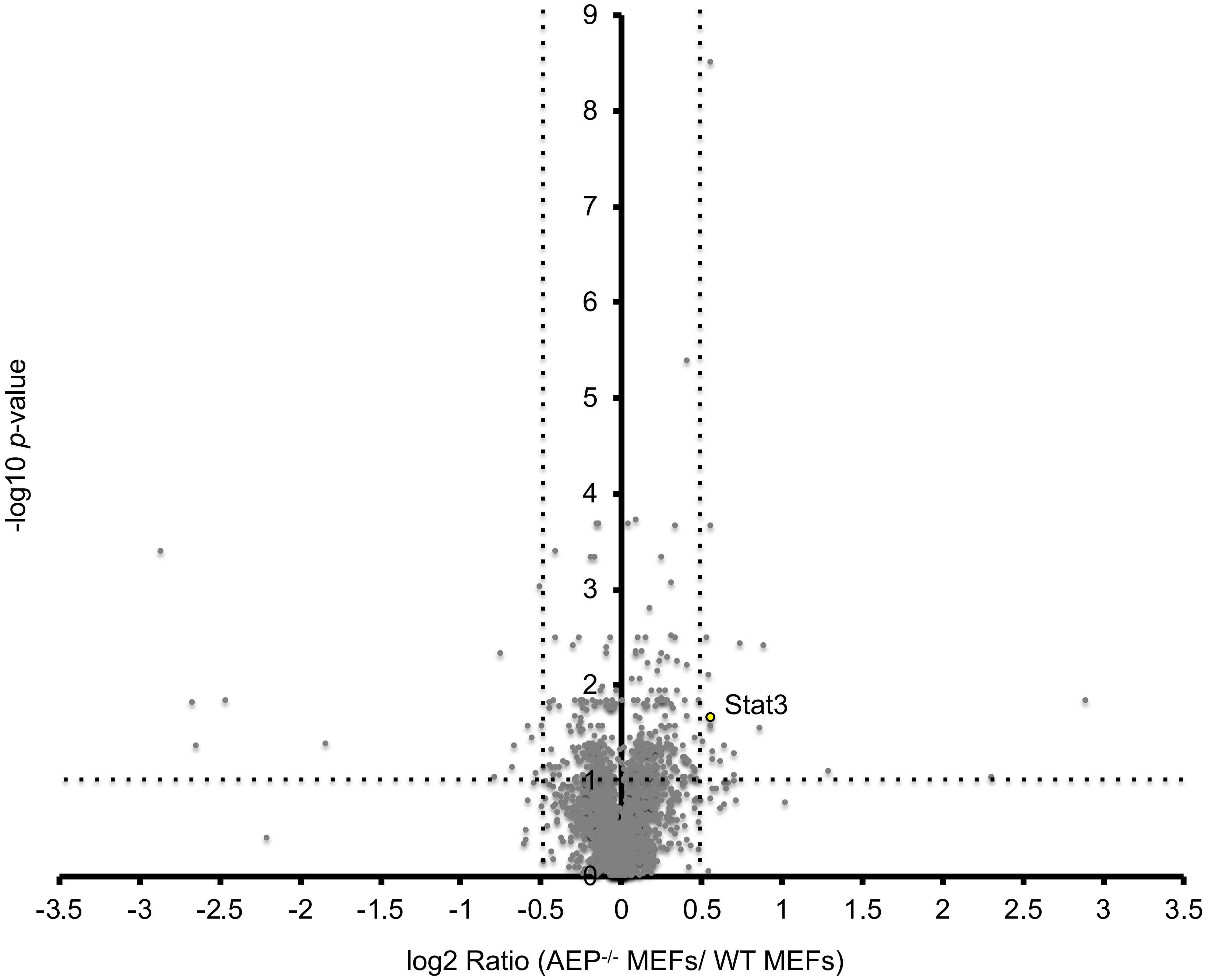

b

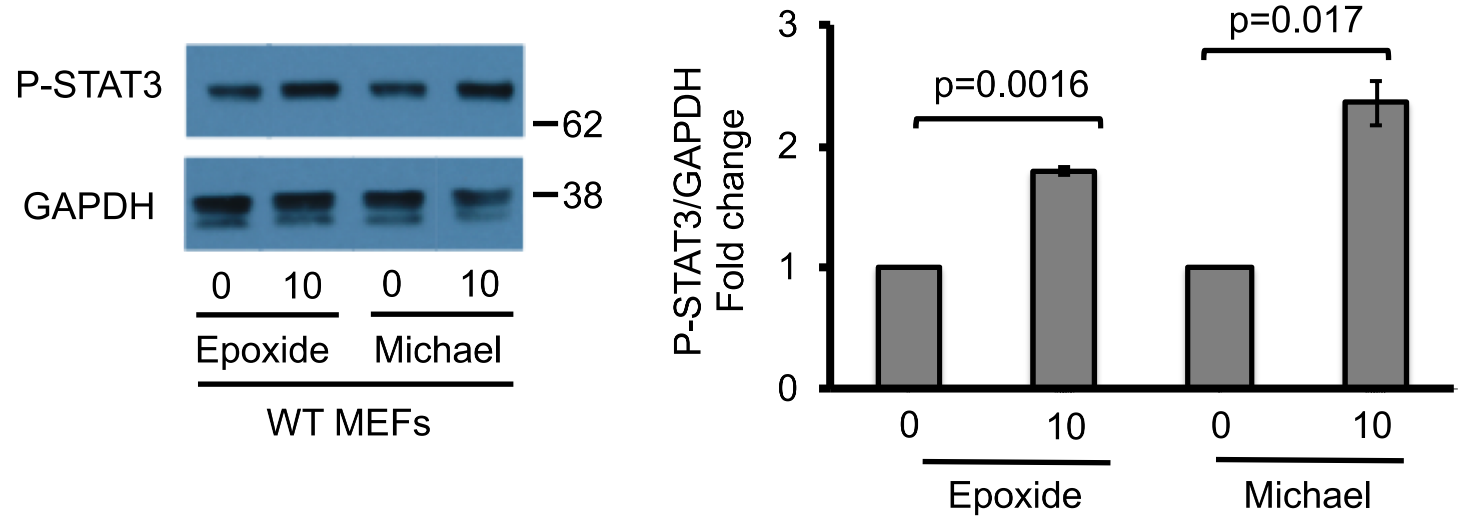

Supplementary Figure 4.

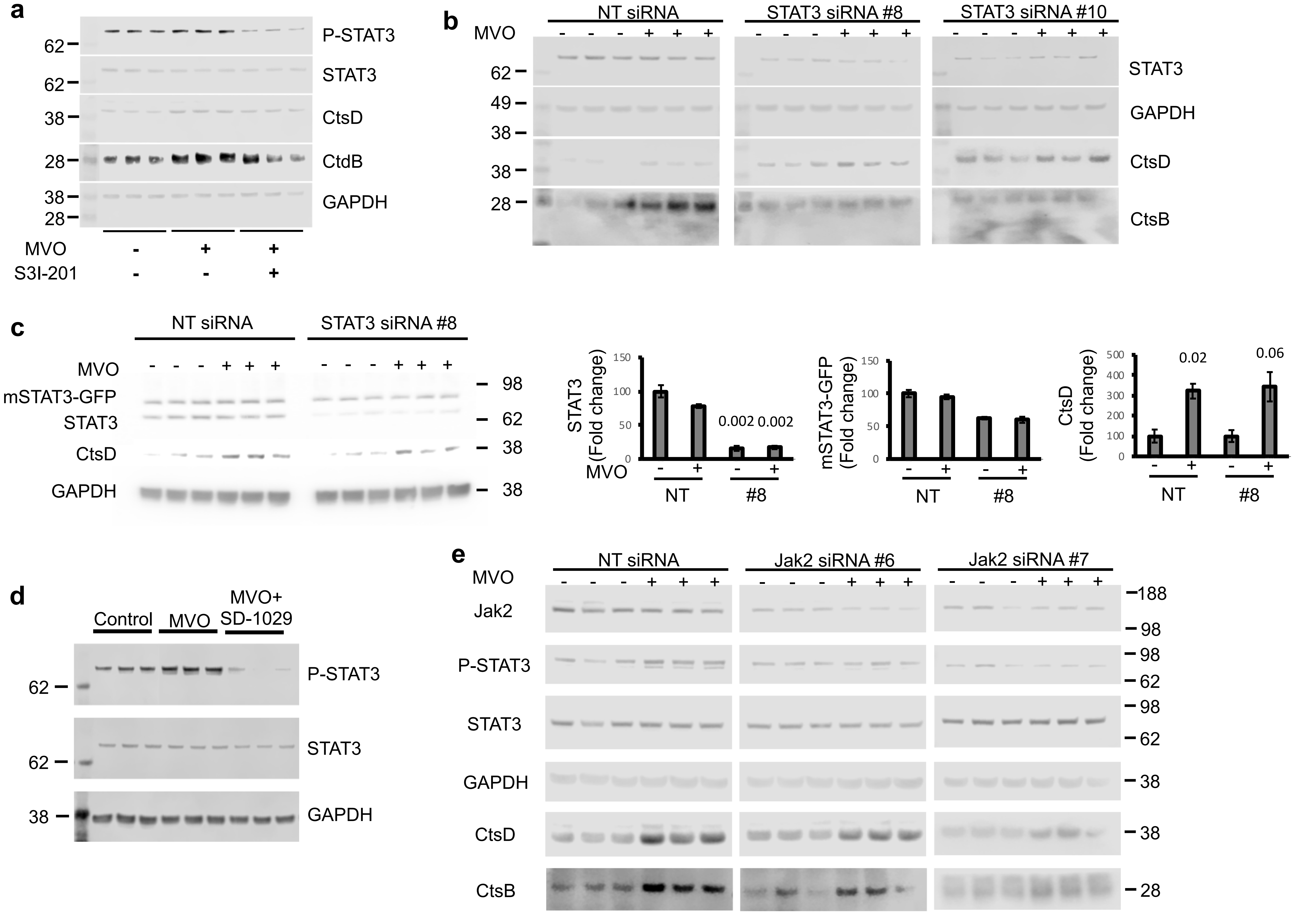

Supplementary Figure 5.

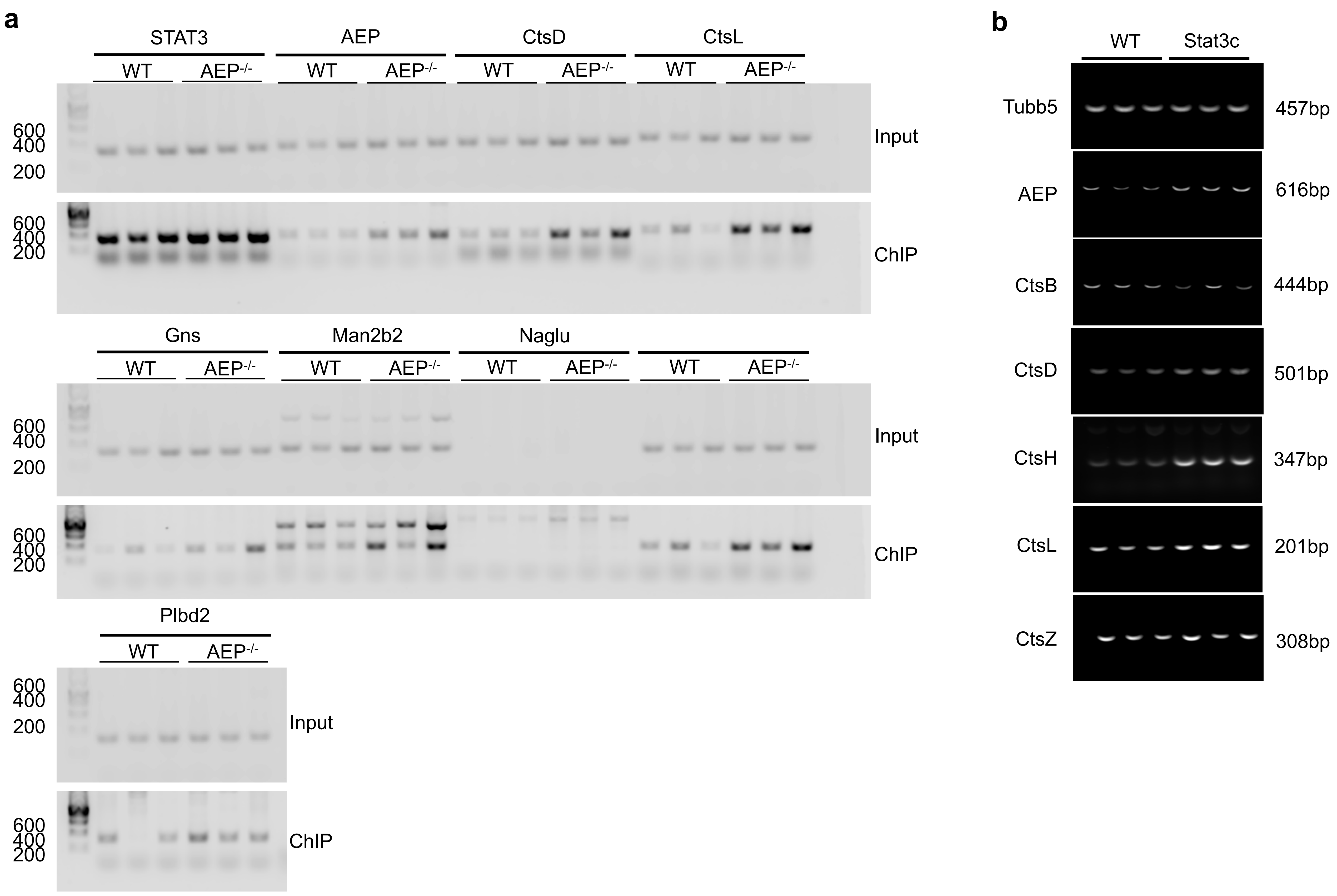

Supplementary Figure 6.

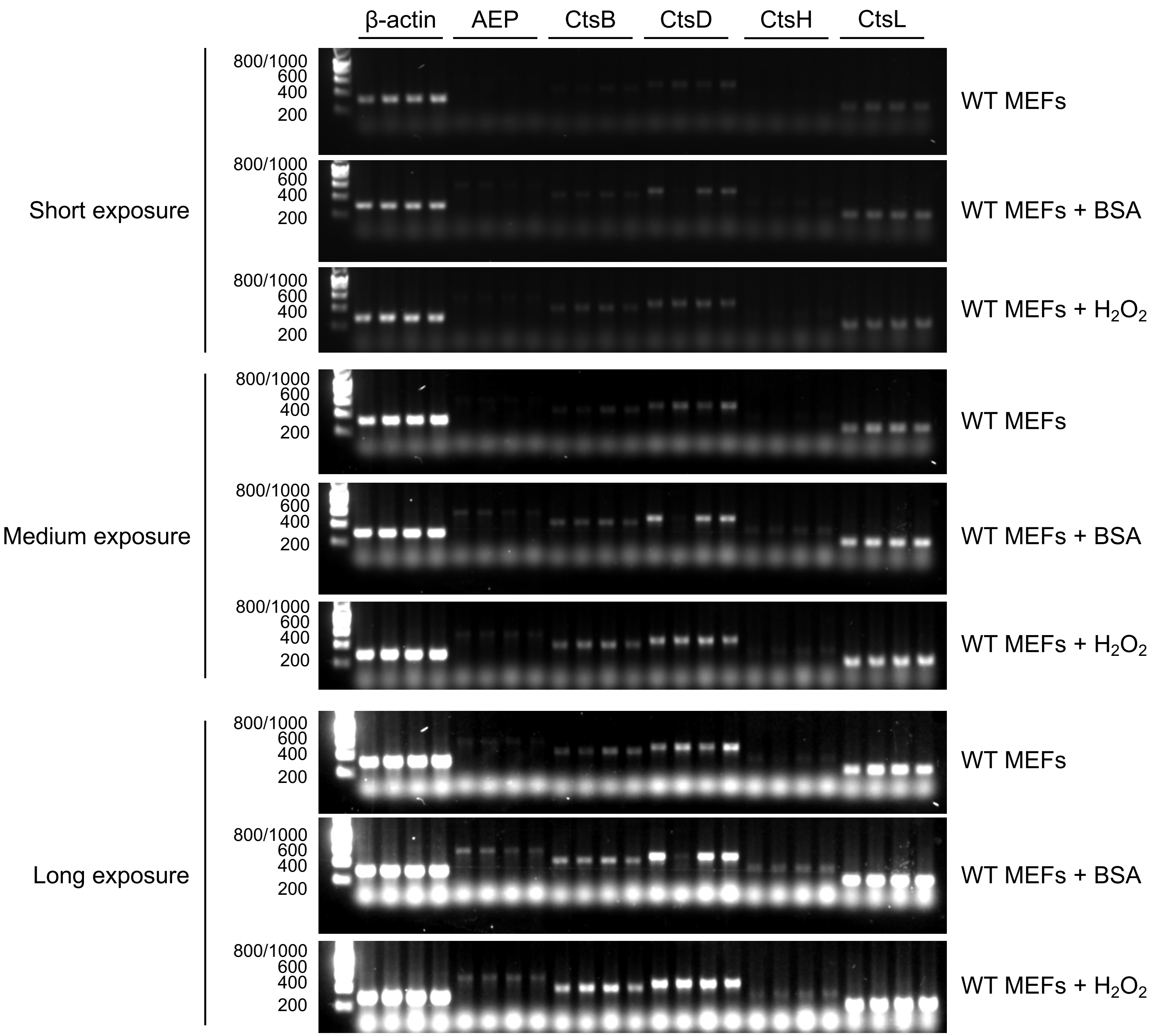

Supplementary Figure 7.

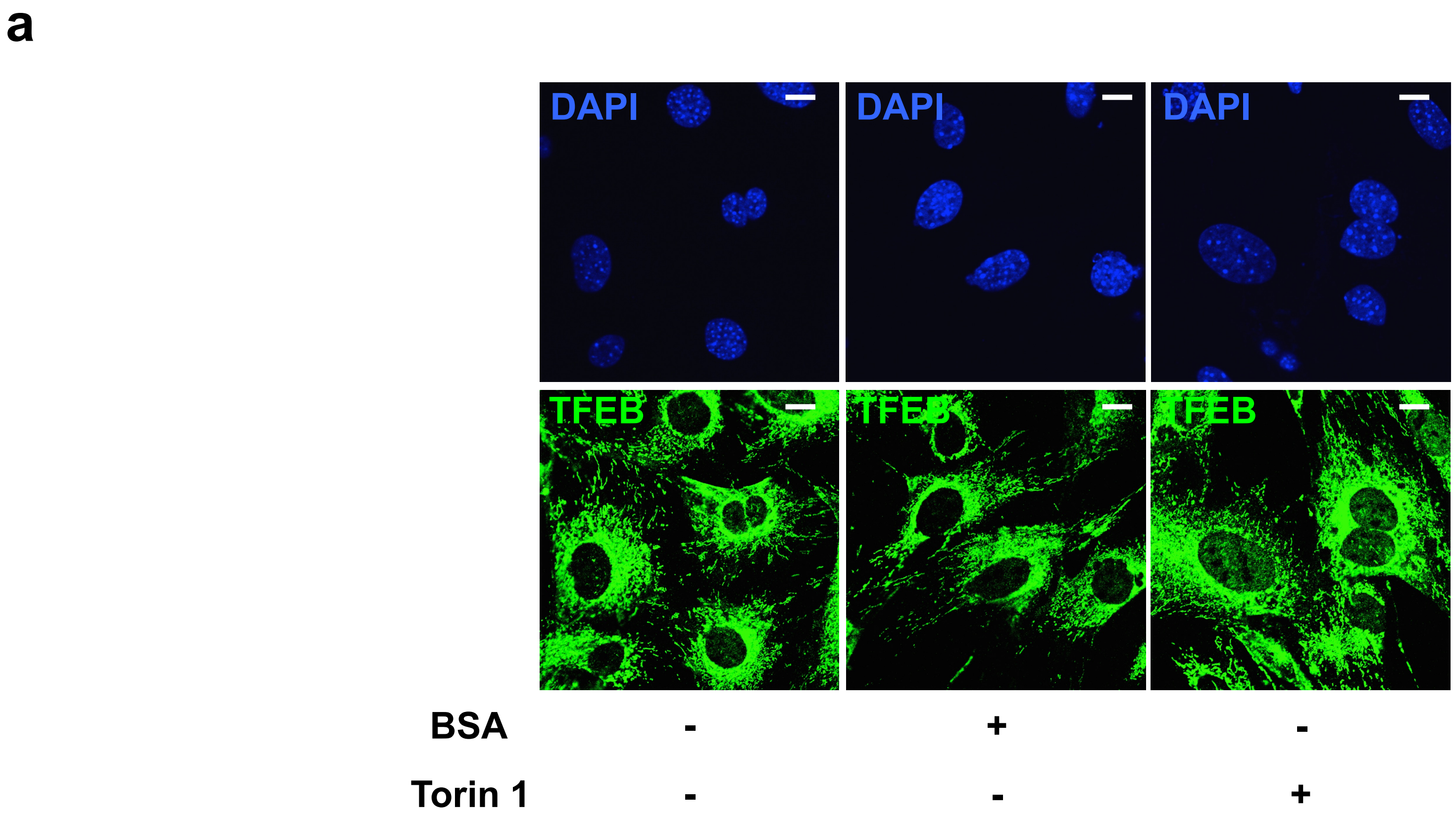

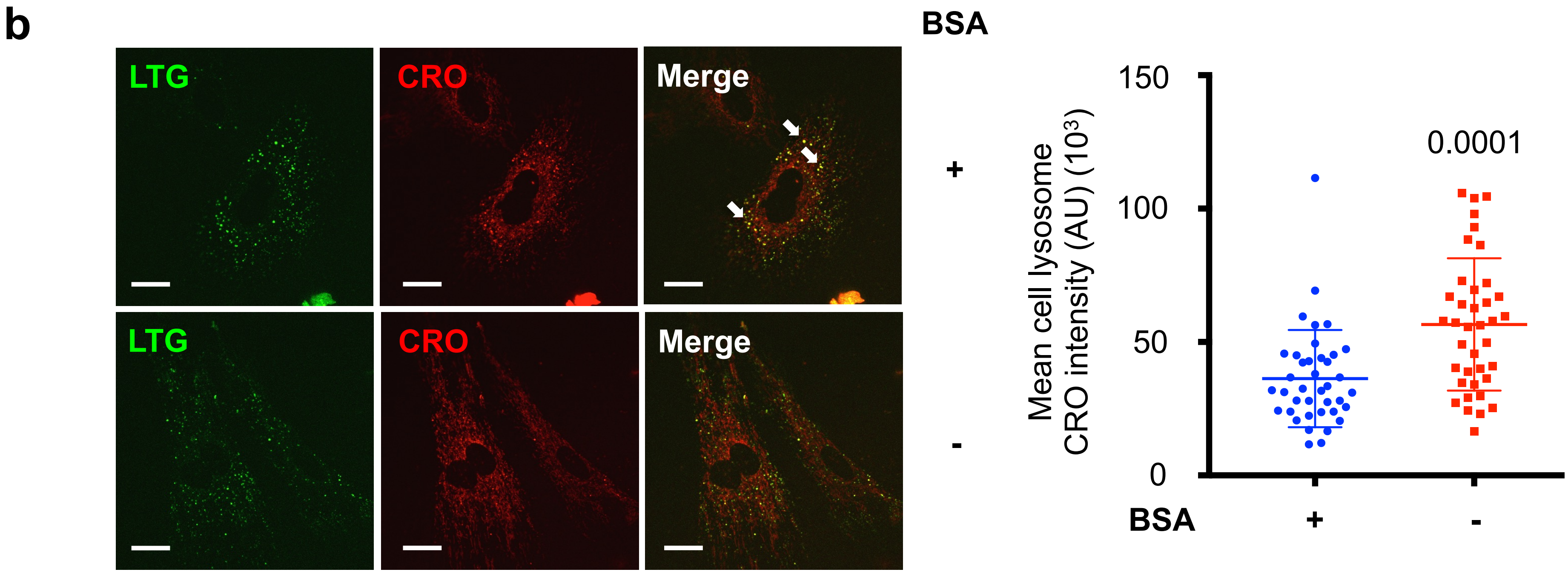

Supplementary Figure 8.

SUPPLEMENTARY FIGURE LEGENDS

Supplementary Figure 1. SILAC-based mass spectrometry analysis of WT vs. AEP$^{-/-}$: proteomics workflow. (a) Lysosomes purified from kidneys obtained from WT mice labelled with L-lysine-^{13}C6 were used as an internal reference sample to analyse by mass spectrometry unlabelled lysosomes purified from WT and AEP$^{-/-}$ kidneys. **(b)** WT MEF (normal Arg & Lys; R0 K0) or AEP$^{-/-}$ MEF (heavy labelled Arg & Lys; K6 R10) were mixed in a 1:1 ratio, and fractions corresponding to cytoplasm, membrane and nucleus were obtained. The samples were subjected to SDS PAGE, each gel lane was cut into 0.5 cm slices and these were analysed by nanoLC-MS/MS. **(c)** *In vivo* activity of cathepsins L in WT and AEP$^{-/-}$ MEF using Magic Red Cathepsin L detection. (Scale bar= 20µm).

Supplementary Figure 2. SILAC-based mass spectrometry analysis of the membranous fraction of WT MEFs vs. WT MEFs+MVO26630. (a,b) Volcano plots showing proteins under-represented (negative log2 Ratio) and over-represented (positive log2 Ratio) in the membranous fraction of WT MEF treated with 50µM MVO26630 for 12 hours **(a)** and 48 hours **(b)** compared to WT MEF against the -log10 *p*-value (two-sided unpaired t test) for three independent experiments.

Supplementary Figure 3. TFEB knockout generation in HKC-8 cells by Crispr-Cas9 targeting two different exons. (a) Immunoblot for TFEB in individual clones of targeted HKC-8 cells. **(b)** Effect of starvation-induction on CtsB levels in HKC-8 WT and two different HKC-8 TFEB knockouts Data are the average ± SD of n≥3. Statistical significance was calculated using a two-sided unpaired t test. **(c)** Gels analysis of the mRNA levels for different lysosomal proteases in WT and TFEB Kos HKC-8 cells treated or untreated with 50µM MVO26630 for 16 hours.

Supplementary Figure 4. Volcano plots corresponding to the cytoplasmic fraction of wildtype and AEP$^{-/-}$ MEF. (a) Volcano plot showing proteins over-represented or under-represented in the cytoplasmic fraction of AEP$^{-/-}$ compared to WT MEF against the -log10 *p*-value (two-sided unpaired t test) for three independent experiments. **(b)** Levels of P-STAT3 in WT MEF treated for 16h with increasing concentrations of mechanistically distinct AEP

inhibitors. Relative intensity of each P-STAT3 band was calculated for 3 independent experiments. Statistical significance was calculated using a two-sided unpaired t test.

Supplementary Figure 5. **Regulation of cathepsin expression by Jak2/STAT3 signalling (a)** Immunoblot analysis of CtsD, CtsB and P-STAT3 in WT MEFs compared to WT MEF treated overnight with 50µM MVO26630 with or without 100µM S3I-201. **(b)** Immunoblot analysis of CtsD, CtsB and STAT3 in HKC-8 WT cells transfected with non-targeting siRNA (NT siRNA) or with two different STAT3-targeting siRNAs (#8 and #10) treated or not with 50µM MVO26630 for 16h. **(c)** Immunoblot analysis of CtsD and STAT3 in HKC-8 cells transiently transfected with an siRNA resistant form of STAT3 and challenged with a non-targeting siRNA (NT siRNA) or a STAT3-targeting siRNAs (#8) prior to treatment with or without 50µM MVO26630 for 16h. Data are the average ± SD of n=3. Statistical significance was calculated using a two-sided unpaired t test. **(d)** Immunoblot analysis of P-STAT3 and STAT3 in HKC-8 WT cells treated overnight with 50µM MVO26630 with or without 10µM SD-1029. **(e)** Immunoblot analysis of Jak2, P-STAT3, STAT3, CtsD and CtsB in HKC-8 WT cells transfected with non-targeting siRNA (NT siRNA) or with two different Jak2-targeting siRNAs (#6 and #7) treated or not with 50µM MVO26630 for 16h.

Supplementary Figure 6. STAT3 ChIP analysis and mRNA levels in STAT3c cells (a) Agarose gel analysis of the ChIP-PCR products for STAT3, AEP, CtsD, CtsL, Gns, Man2b2, Naglu and Plbd2 in WT and AEP deficient MEFs. **(b)** Agarose gel analysis of the mRNA levels encoding several different lysosomal proteases in wildtype and STAT3c 3T3 cells.

Supplementary Figure 7. mRNA levels in WT MEF treated with BSA or H2O2 Agarose gel analysis of the mRNA levels encoding several different lysosomal proteases in WT MEFs treated or not with BSA 30mg/ml or with H_2O_2 0.2mM overnight.

Supplementary Figure 8. Lysosomal protein overload does not affect TFEB cytoplasmic localisation but induces lysosomal oxidative stress. (a) Lysosomal protein overload (30 mg/ml BSA for 16h) does not induce nuclear relocalisation of TFEB, while mTOR inhibition with Torin1 (1µM) drives the nuclear translocation of TFEB. (Scale bar= 20µm). **(b)** Live cell

imaging of lysosomal production of reactive oxygen species using CellROX Orange (CRO) in WT MEF treated or not with 30 mg/ml BSA for 16h. LysoTracker Green DND26 (LTG) was used as a lysosomal marker. Data are means ± SEM calculated using 20 cells per experiment (Scale bar= 10µm). Statistical significance was calculated using a two-sided unpaired t test.
