# Supplementary material for: Lysosomal protease deficiency or substrate overload induces an oxidative-stress mediated STAT3-dependent pathway of lysosomal homeostasis
Source: Nat Commun. 2018 Dec 17;9:5343. doi: 10.1038/s41467-018-07741-6 (PMC6297226; doi:10.1038/s41467-018-07741-6)
Supplement: Supplementary file 2 — Peer Review File [file 41467_2018_7741_MOESM2_ESM.pdf]

Editorial Note: this manuscript has been previously reviewed at another journal that is not operating a transparent peer review scheme. This document only contains reviewer comments and rebuttal letters for versions considered at Nature Communications.

Reviewers' Comments:

Reviewer #1 (Remarks to the Author):

In my opinion, the authors have addressed Reviewer 1's concerns satisfactorily. This is a very nice piece of work that puts forward a novel concept (role of STAT3 in lysosomal adaptation) and is supported by excellent data and multiple independent lines of evidence.
